# Age-related differences in subjective and physiological emotion evoked by immersion in natural and social virtual environments

**DOI:** 10.1038/s41598-024-66119-5

**Published:** 2024-07-03

**Authors:** Katarina Pavic, Dorine Vergilino-Perez, Thierry Gricourt, Laurence Chaby

**Affiliations:** 1 Université Paris Cité, Vision Action Cognition, F-92100 Boulogne-Billancourt, France; 2 SocialDream, Research and Development Department, Bourg-de-Péage, France; 3 Sorbonne Université, Institut des systemes intelligents et de robotique (ISIR), CNRS, F-75005 Paris, France

**Keywords:** Human behaviour, Geriatrics

## Abstract

Age-related changes in emotional processing are complex, with a bias toward positive information. However, the impact of aging on emotional responses in positive everyday situations remains unclear. Virtual Reality (VR) has emerged as a promising tool for investigating emotional processing, offering a unique balance between ecological validity and experimental control. Yet, limited evidence exists regarding its efficacy to elicit positive emotions in older adults. Our study aimed to explore age-related differences in positive emotional responses to immersion in both social and nonsocial virtual emotional environments. We exposed 34 younger adults and 24 older adults to natural and social 360-degree video content through a low immersive computer screen and a highly immersive Head-Mounted Display, while recording participants' physiological reactions. Participants also provided self-report of their emotions and sense of presence. The findings support VR’s efficacy in eliciting positive emotions in both younger and older adults, with age-related differences in emotional responses influenced by the specific video content rather than immersion level. These findings underscore the potential of VR as a valuable tool for examining age-related differences in emotional responses and developing VR applications to enhance emotional wellbeing across diverse user populations.

## Introduction

While aging is often associated with social isolation and withdrawal^1^, older individuals also tend to prioritize emotional well-being and report high levels of life satisfaction^2,3^. Moreover, they exhibit a greater inclination to process positive rather than negative information^4^, which may facilitate the maintenance of their social and emotional well-being^5,6^. This age-related tendency to favor the processing of positive over negative information is known as the ‘positivity effect’^4^. Although extensive research has explored how the positivity effect impacts information processing (for a review see Ref.^7^), it remains unclear whether this positivity effect extends to emotional reactivity^8,9^. Furthermore, there has been limited attention regarding how older adults experience positive emotion in real-life situations^10,11^. The emergence of Virtual Reality (VR) has provided a unique opportunity to investigate emotional processes in ecologically valid environments while maintaining high experimental control^12^. However, current studies on the processing of positive emotion in VR often lack social context, primarily relying on the utilization of natural environments (for a review see Ref.^13^). Therefore, there is a compelling need to integrate social contexts within VR research and to utilize a comprehensive approach by simultaneously examining subjective and physiological variables to capture the complexity of emotional responses in older adults.

With advancing age, an “emotion paradox” emerges as older adults' tend to exhibit increased positivity and life satisfaction, despite typically confronting physical decline and social losses. A key theoretical framework to understand this phenomenon is the socioemotional selectivity theory (SST^14^). According to SST, as individuals age and become more aware of their limited remaining lifetime, they prioritize goals related to social connections and emotional well-being. This motivational shift is often manifested as a positivity effect, characterized by a bias towards processing positive emotional information and minimizing negative information^4^. However, this positivity effect does not seem to imply an increased emotional response to positive information in older adults. In fact, previous studies have yielded inconsistent evidence regarding age-related changes in emotional reactivity to positive emotional stimuli^15^, with some reporting an increase^16–18^, others indicating no age-related differences^19,20^ and some even suggesting a dampening^21–23^ of older adults’ emotional responses. The mixed results observed may stem from the methods used for measuring emotional response and/or the material employed to induce positive emotions. This study aims to explore these factors in greater detail.

Combining self-reported and physiological measures has been considered necessary for obtaining a comprehensive understanding of age-related changes in emotional responses^24,25^. However, several studies have documented that, with advancing age, there is a disconnection between peripheral physiological responses and the subjective experience of events (for a review see Ref.^26^). This phenomena, termed as ‘maturational dualism’^26^, is reflected opposite age-related differences in subjective and physiological responses to the affective material. Regarding subjective emotional responses, which are commonly assessed through self-reports, older adults tend to report experiencing more positive emotions and higher levels of arousal compared to younger adults when exposed to the same affective material^16–18,25^ (although see Ref.^27^ for contrasting findings). In contrast, older adults generally show attenuated physiological responses (i.e., heart rate and skin conductance) compared to younger adults^20,25,28,29^. Nevertheless, a thorough investigation of the temporal dynamics of physiological responses during exposure to affective material would significantly expand our comprehension of age-related changes in emotion processing^6,30^.

As the majority of studies investigating age-related changes in emotional responses primarily relied on brief presentations of standardized stimuli in lab-based conditions they may lack ecological validity^11,31^. The use of dynamic and/or multimodal stimuli has been shown to provide a more accurate representation of emotional processes as they occur in everyday life^32–34^. Thus, materials with higher ecological validity are crucial for gaining insight into how age-related differences in positive emotion processing occur and unfold in real-life contexts. In recent years, VR has emerged as a powerful tool for studying emotional processes^35,36^. Complementing traditional emotion induction procedure that involves presenting affective pictures^37^, videos^38^, sentences^39^ or music^40^, VR offers unique advantages for emotion induction due to its immersive characteristics and the sense of presence it elicits^36^. Immersion refers to the objective properties of a device to deliver multisensory simulations similar to those experienced in real life^41^. The sense of presence encompasses both the subjective feeling of "being physically there" in the virtual environment (spatial sense of presence^42^) and the feeling of "being virtually there with others" (social sense of presence^43^). These key features of VR contribute to eliciting emotional responses that are close to those encountered in real-life environments^44,45^.

Surprisingly few studies have relied on VR to investigate older adults’ emotional responses to positive information^46–50^. These studies primarily aimed to determine the efficacy of VR to induce positive emotions and improve well-being in older adults. Although initial results indicate VR's effectiveness in eliciting positive emotions^46–50^, there has been insufficient exploration of how emotional responses may vary with advancing age. Additionally, the use of predominantly self-reported measures to assess emotional responses potentially limited the robustness of these results. To our knowledge, only one study^46^ has assessed both subjective and physiological responses of older adults to a VR experience. Its findings indicated that older adults manifested increased positive emotions on self-reports following exposure to the VR experience, in contrast to attenuated physiological responses during exposure to VR. However, as this study exclusively focused on older adults, its findings hinder the understanding of age-related differences in emotional responses to positive information. Another major limitation of this literature is the interchangeable use of the term “VR” to refer to highly immersive Head-Mounted Displays (HMDs)^46,48–50^ and to less immersive devices such as computer screens^47^. While it is generally accepted that highly immersive VR devices are more effective at eliciting positive emotions on both subjective and physiological levels in younger adults^51,52^, their superiority in eliciting positive emotions in older adults remains inconclusive^50,53^. Moreover, the use of HMDs can present additional challenges with older adults, as they often exhibit a negative attitude towards novel technologies^54^, prefer less immersive devices^53^, and express concerns regarding the physical discomfort associated with the use of immersive devices^55,56^. Therefore, determining the optimal level of immersion required to induce positive emotions in older adults is crucial before fully addressing the potential of VR for studying age-related differences in emotional responses. To the best of our knowledge, no studies have directly compared the efficacy of highly immersive HMDs with less immersive computer screens for eliciting positive emotions in older adults. In addition, fewer studies have examined age-related differences in sense of presence, although it can contribute to emotion induction with VR^51,57^.

Lastly, the influence of the content of the affective material on emotional responses has not been sufficiently explored neither in traditional lab-based studies^58,59^ nor in VR-based settings^13,60^. Specifically, the influence of social contents, particularly involving social interactions on emotional responses remains largely unexplored. Yet, traditional non-VR studies consistently demonstrate that social affective contents evokes higher levels of subjective and physiological arousal compared to non-social contents in younger adults^58,61,62^. While to our knowledge no study has directly addressed the influence of stimuli content on older adults’ emotional responses, one study^18^ revealed that pleasant pictures featuring babies or families elicited increased self-reported arousal in older adults. Despite the advantages offered by VR to examine emotional responses to social scenarios^63^, VR-bases studies have predominantly relied on natural landscapes (i.e., non-social affective contents), revealing that exposure to virtual nature elicits a relaxed state in younger^46,64–66^ and older adults^46,49,67^. Fewer studies have so far utilized social contents in VR^49,51,65^ and provided mixed results regarding the effects of social contents on emotional responses: while immersion in social contents seem to elicit greater subjective and physiological arousal compared to natural landscapes in younger adults^51^ (although see Ref.^65^), older adults manifested greater subjective arousal following immersion in virtual nature compared to social contents^49^. However, none of the above cited studies have compared age-related differences in emotional responses evoked by social and nonsocial contents, as they involved either younger or older adults. Thus, the specific impact of social and non-social affective contents on emotional responses remains to be thoroughly explored in aging.

The present study aims to provide valuable insights into the complex interplay between age, immersion levels, and content of the affective material in shaping positive emotional experiences. Regarding self-reports, we expect significant age-related differences, with older adults likely to report more positive ratings and higher arousal compared to younger adults. These differences are anticipated to be more pronounced with stimuli presented on the less immersive device compared to more immersive one. Concerning stimuli content, we posit that age-related differences will be more pronounced for nonsocial than social contents.

In terms of physiological responses, measured by heart rate and skin conductance levels, we predict older adults will exhibit less pronounced reactions than younger adults. We further anticipate that the age-related difference in physiological responses will be more pronounced following exposure to highly immersive device compared to the less immersive one. Regarding stimuli content, we expect more pronounced age-related differences for social contents than social contents.

Finally, we explored the hypothesis that the sense of presence, influenced by the level of immersion and the type of content, could lead to differences between age groups. While it is generally assumed that highly immersive devices enhance the sense of presence in young adults, our objective is to investigate whether older adults experience this effect to the same extent as younger adults, particularly when they are exposed to social versus natural content.

## Methods

### Participants

Initially, 65 participants were recruited, including 38 young adults and 27 older adults. Inclusion criteria required participants to have no history of psychiatric, neurological or cognitive impairment. All participants needed to have normal or corrected-to-normal vision and hearing. Older participants were also required to score above the 27/30 cutoff on the Mini Mental State Examination (MMSE^68^). Data from three older adults were not included in the analyses due to either having a MMSE score below the cut-off or misunderstanding the instructions, resulting in numerous missing data. Additionally, four younger adults were not included in the analyses based on outlier detection of their physiological data in the control condition.

Thus, the final sample consisted of 34 young (17 women, 17 men, mean age 22.21 ± 1.86) and 24 elderly participants (16 women, 8 men, mean age 69.92 ± 5.95). Participants characteristics are reported in Table 1. All participants had at least a minimal technology proficiency, determined by their ability to use a mouse and their daily use of at least one digital device. Additionally, we ensured that none of the participants owned an HMD.

**Table 1:** Sample characteristics.

	Younger adults (n = 34)	Older adults (n = 24)	Age-related differences
	Mean ± SD	Mean ± SD	*p*-value
Demographic			
Age (years)	22.21 ± 1.86	69.92 ± 5.95	** < 0.001**
Gender (men:women)	17:17	8:16	0.21^a^
Education (years)	15.56 ± 1.05	13.75 ± 2.97	0.06^b^
Previous VR experience (yes:no)	22:12	12:12	0.26^a^
Cognitive functionning			
MMSE	–	28.96 ± 1.08	–
Affective state			
HADS anxiety	9.56 ± 4.45	6.92 ± 4.27	**0.03**
HADS depression	3.82 ± 2.81	3.50 ± 2.41	0.65

Significant values are in bold.

a Chi-square tests.

b Mann-Whitney U-test.

A power analysis was conducted with the PANGEA Shiny App^69^ to estimate the sample size required to detect at a 80% power medium effect sizes, based on similar research studies that induced emotions in younger and older adults using videos^24,70^. For our mixed factor design, with Age-group as a between-subject factor, Immersion and Content as within-subject factors, by individual random effects with 2 repetitions and an estimated effect size of d = 0.30 at least 24 participants per age-group are required to achieve a power of 0.80.

This research was conducted in accordance with the Declaration of Helsinki and approved by the Ethics Committee of Université Paris Cité (IRB No. 00012021-61). All participants provided written informed consent prior to the study and received a financial compensation of 15 euros for their participation.

### Stimuli

**Figure 1:**
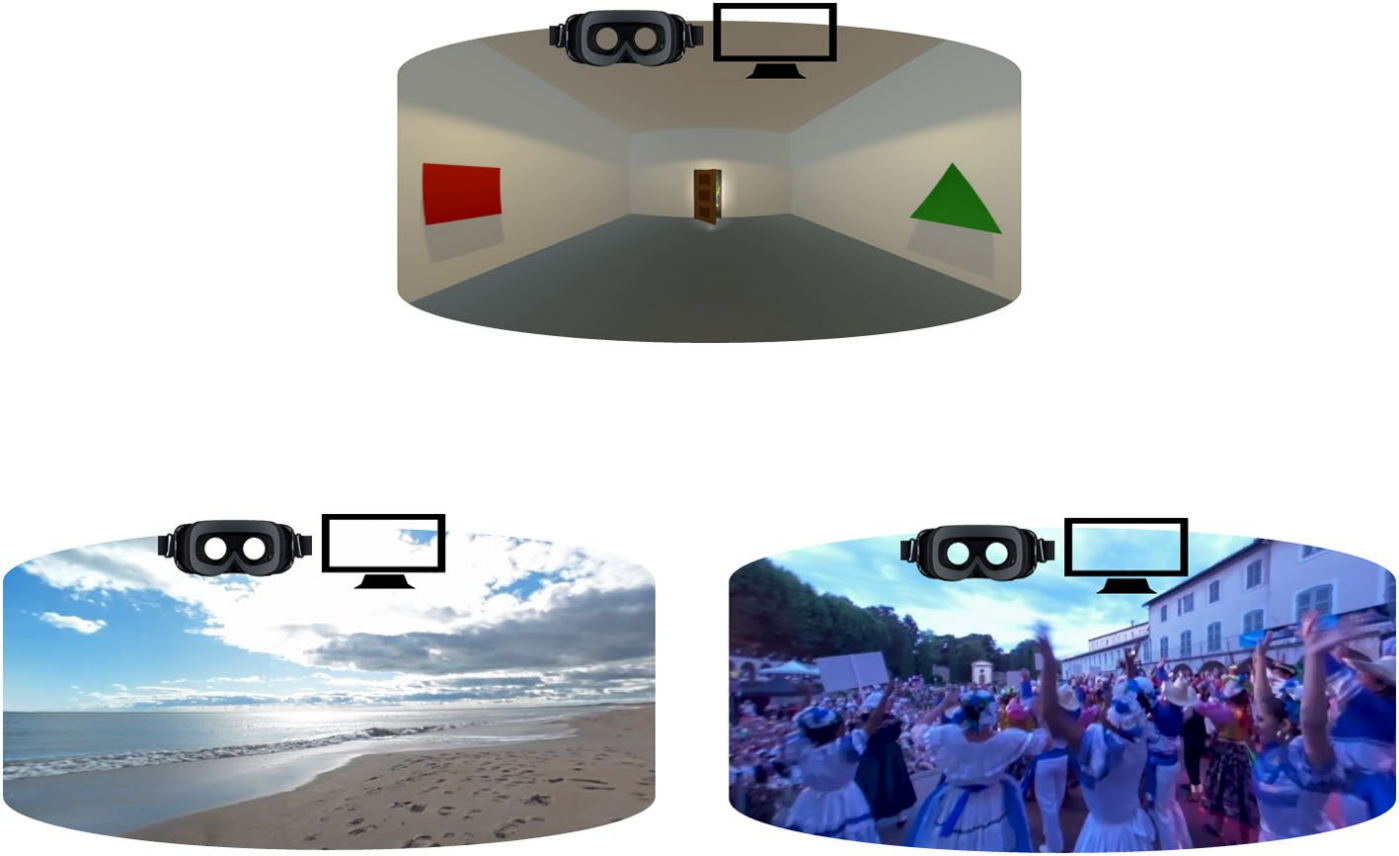
Illustration of the experimental material.

For the present study, we elaborated a collection of nine 360-degree videos, comprising a control video with neutral emotional valence, and eight videos specifically designed to induce positive emotions (Fig. 1 provides an illustration of the videos). All the videos were pre-tested for inducing the target neutral or positive emotions in 10 younger and 10 older adults. The pre-test was designed to ensure they effectively induced the intended emotions and were as pleasant and acceptable across age groups. Individuals who participated in the pre-test were not eligible for the main study to prevent preconditioning bias. Note that the efficacy of the selected video contents to induce the target emotions in younger adults was additionally validated in a previous study^51^.

All videos had a high resolution of 4 K and were accompanied by corresponding sound based on the context. The neutral control video was developed using Unity 2021.1.0 and depicted an empty room with shapes on the wall and a slightly open door. The eight 360-degree videos designed to induce positive emotions consisted of real places or events captured with a GoPro Fusion 360 camera. Half of these videos presented natural content, highlighting vegetation or aquatic features such as the sea or cascades. The other half featured social content, depicting people in various settings, such as taking a stroll or attending a concert. A portion of the control video content was used in the first instance to familiarize participants to viewing 360-degree videos and to navigate within them. After this familiarization phase ended, participants were immersed in two-minute scenarios categorized as either control, natural, or social. Before each scenario, a 10-s black screen served as a buffer phase. Data from the buffer and familiarization phases were not included in the analysis. Movement within the videos was facilitated using a technique referred to as “teleportation motions”, which involves changes in the viewer’s perspective with visual transition or “jumps” from one location to another without simulating linear motion^71^. Participants experienced a total of 6 “teleportation” every 20 s throughout the video duration. Such a motion strategy has been recognized as effective in minimizing the incidence of cybersickness^71,72^.

To compare levels of immersion, two devices were utilized. A computer screen (25-inch Iiyama screen, 1920 × 1080 pixels resolution) served as a weakly immersive device, while an HMD (Samsung HMD Odyssey +, 1440 × 1600 pixels resolution) was employed as a highly immersive device. On the low-immersive device (i.e., the screen), participants could explore the 360-degree video contents with mouse movements, while in the highly immersive condition they could explore the video contents by head movements. Figure 1 illustrates the material employed for the present study.

### Measures

Anxious and/or depressive symptomatology in the last seven days was assessed using the Hospital Anxiety and Depression Scale (HADS^73^). As shown in Table 1, younger adults exhibited significantly higher levels of anxiety compared to older adults. There were no significant age-related differences in the depressive symptomatology. Additionally, cognitive impairments in the elderly participants were assessed using the MMSE.

Emotional responses were evaluated through a combination of self-report questionnaires and physiological measures. Self-reported valence and arousal ratings were obtained using the Self-Assessment Manikin (SAM^74^). Positive affect (excitement, joy, relaxation, interest) and negative affect (anxiety, anger, sadness, boredom) were assessed using 7-point Likert scales.

Physiological emotional responses, including heart rate (HR, beats-per-minute) and electrodermal activity (EDA, µSiemens), were captured continuously throughout the session using the Empatica E4 wristband, capturing HR at 1 Hz and EDA at 4 Hz. Visual inspections were conducted on the HR data to identify any failed measurements. Signal drops were excluded to avoid downwardly biasing averages^75^. The EDA data were processed and analyzed using the LEDALAB V3.4.9. toolbox, run in Matlab 2017b. This process involved visual check for artifacts and their subsequent correction. Continuous Decomposition Analysis was applied to the cleaned signal to extract the Skin Conductance Level (SCL). Due to notable variability and skewness in SCL values across participants, the range of each participant’s SCL was computed using the following formula^66,76^:

$$S= \frac{\text{s }-\text{min}(s*)}{\text{max }(\text{s}*) -\text{ min }(\text{s}*)}$$

In the above formula, *s* reflects the raw value of a participants’ SCL at a specific time during the session, s^*^ represent the SCL signal over the entire session for that participant, and *S* represents the normalized value (in percentages) of the SCL signal.

For both HR and SCL data, outliers that were ± 3 SD from the mean of each video content per age group and immersion level were removed from the analyses.

To assess the sense of presence, two additional self-report questionnaires were used. The Spatial Presence Experience Scale (SPES^77^) was used to measure the spatial sense of presence and the the Social Richness subscale of the Temple Presence Inventory (TPI-SR^78^) was used to measure social Sense of Presence.

### Procedure

The procedure employed in this study closely followed the methodology outlined in a previously published study^51^. Participants were exposed to control, natural, and social video contents using both a highly immersive HMD and a less immersive screen. The immersion levels were counterbalanced between participants. The experimental session began with a training phase within the control video environment, allowing participants to familiarize with the virtual setting and navigation tools—head movements for the HDM or mouse movements for the screen—required for exploration. After this familiarization, we recorded participants’ physiological responses during the two-minute control video. Participants then engaged with four videos, consisting of two natural and two social videos, presented in a randomized order. After completing the viewing session on one device (i.e., one control, two natural and two social video contents), participants switched to the second device and repeated the same procedure. Throughout this process, participants were encouraged to engage with the videos as naturally as possible and explore the virtual environments at the pace that felt the most comfortable for them. Following each video, participants provided self-reports of their valence, arousal, positive and negative affect, and spatial and social sense of presence. Simultaneously, physiological measurements were collected throughout the entire procedure, which lasted approximately one hour.

### Statistical analyses

ANOVA analyses were conducted on self-reported emotional responses, as well as spatial and social senses of presence. These analyses were computed on R 4.3.0^79^, with the *afex* package. When necessary, Greenhouse–Geisser corrections were employed if the sphericity assumptions were not met. For clarity’s sake, we report uncorrected degrees of freedom for the analyses concerned. Post-hoc analyses with Bonferroni corrections for multiple comparisons were further performed when relevant.

To specifically capture the dynamic changes over time in HR and SCL while participants watched the control, non-social and social video contents on both levels of immersion, we segmented the data into six time-bins of 20 s each (0–20 s, 20–40 s, 40–60 s, 60–80 s, 80–100 s and 100–120 s). This approach allowed us to compute average HR and SCL values for these intervals, which were subsequently entered into our Growth Curve Analyses (GCA)^80^. GCA is well-suited for analyzing time-series data, as it employs polynomial functions to quantify the slope and inflection of data over time. Specifically, the 'linear term' estimates the slope, the 'quadratic term' assesses the curvature, and the 'cubic term' is utilized to identify the inflection points of data over time.

We conducted GCA with the *lme4*^81^ package, and statistical significance was assessed using the *lmerTest*^82^ package. HR data were modeled with third-order orthogonal polynomials (including linear, quadratic and cubic polynomials). Normalized SCL data were modeled with second-order orthogonal polynomials (including linear and quadratic polynomials). We selected these time terms based on the visual inspection of the overall shape of the physiological responses time course. We conducted model comparisons to confirm that both models fit the data by adding successively higher-order time terms (i.e., intercept only, linear, quadratic and cubic terms) and testing them for improved fit over the previous model using chi-square tests (see Supplementary Material 2 for results of model comparisons). All models included Age-group (younger adults = 1 *vs* older adults = 1), Level of immersion (low = -1 *vs* high = 1 levels of immersion) and video Content (control content = -1 *vs* natural = 1 and control content = -1 *vs* social = 1) as fixed effects, by-participant random effects on all time terms, and participants-by-conditions random effects on all time terms except the cubic one. All models were specified without correlated random effects to help ensure convergence. Main and interaction effects were examined with *F* statistics using the “anova” function of the *lmerTest* package, followed by *t*-tests on individual parameter estimates to evaluate the contrasts of interests. The *lmerTest* package, which was used to compute these analyses, provides p-values in type III anova and summary tables for linear mixed models via Satterthwaite’s degrees of freedom method.

## Results

### Self-reported emotional response

**Figure 2:**
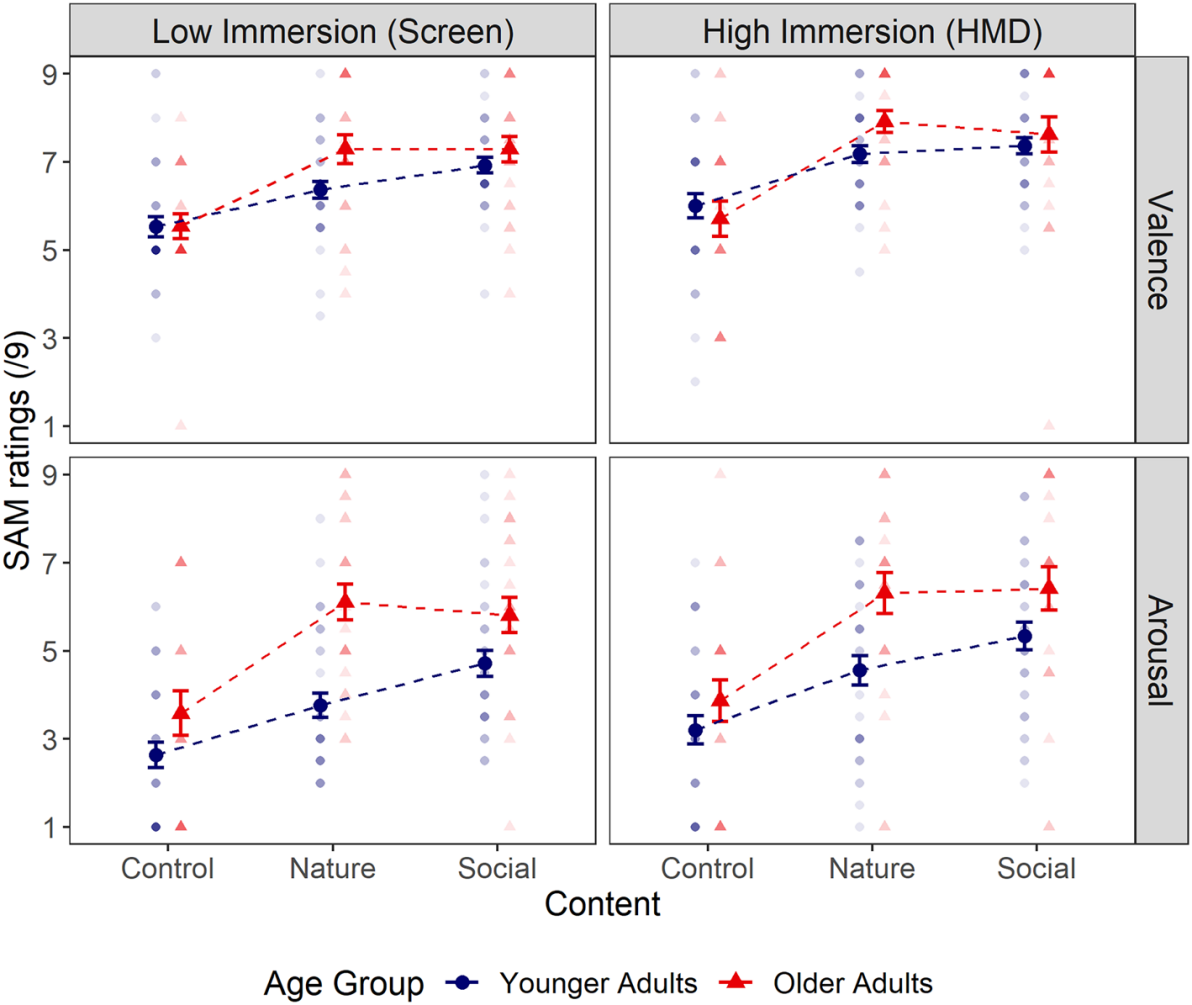
Mean valence and arousal SAM scores reported by younger and older adults for each level of immersion and content. Error bars indicate standard errors from the mean. Points represent individual responses; their brightness indicates the number of participants giving the same rating (i.e., the darker a point is, the more participants attributed the same score).

Valence and arousal ratings were analyzed with ANOVAs including Age-Group (younger *vs* older adults) as between-subject factor, Level of immersion (low *vs* high) and Video contents (Control *vs* Natural *vs* Social) as within-subject factors. Mean valence and arousal ratings for all experimental conditions are illustrated in Fig. 2.

The ANOVA conducted on valence ratings revealed no main effect of Age-Group (*F* (1, 56) = 1.94*, p* = 0.17, ${\eta }_{p}^{2}$ = 0.03). However, a main effect of Immersion emerged (*F* (1, 56) = 13.98*, p* < 0.001, ${\eta }_{p}^{2}$ = 0.20), indicating that participants reported in general greater valence ratings (i.e., more positive emotions) following exposure to the highly immersive device (*M*_*HMD*_ = 6.95 ± 1.68) than the less immersive one (*M*_*Screen*_ = 6.45 ± 1.47). Contrary to our expectation, the Age-Group x Immersion interaction failed to reach significance (*F* (1, 56) = 0.61, *p* = 0.41). A main effect of video content emerged (*F* (2, 112) = 47.58*, p* < 0.001, ${\eta }_{p}^{2}$ = 0.46), revealing that the social (*M* = 7.28 ± 1.36) and natural (*M* = 7.12 ± 1.36) contents induced more positive emotions compared to the control one (*M* = 5.71 ± 1.57, *p*_*s*_ < 0.001). This effect was further modulated by an Age-Group x Content interaction (*F* (2, 112) = 3.50*, p* = 0.03, ${\eta }_{p}^{2}$ = 0.06). However, post-hoc analyses did not reveal any relevant age-related differences in self-reported valence (all p_s_ > 0.1). Instead, post-hoc analyses confirmed the main effect of content for both age-groups, meaning both younger and older adults reported higher valence ratings for social (respectively, *M*_*YA*_ = 7.15 ± 1.06, *M*_*OA*_ = 7.46 ± 1.69) and natural contents (respectively, *M*_*YA*_ = 6.77 ± 1.19, *M*_*OA*_ = 7.60 ± 1.44) compared to the control one (respectively, *M*_*YA*_ = 5.76 ± 1.48, *M*_*OA*_ = 5.62 ± 1.70, all *p*_*s*_ < 0.01). None of the remaining interactions reached significance (all *p*_*s*_ > 0.1). On a similar note, comparable results were found on self-reported positive and negative affect ratings (see Tables S1–S4 in Supplementary Material 1).

The ANOVA conducted on arousal ratings revealed significant age-related differences (*F* (1, 56) = 11.01*, p* < 0.001, ${\eta }_{p}^{2}$ = 0.17), indicating that older adults reported greater arousal (*M* = 5.35 ± 2.50) compared to younger adults (*M* = 4.04 ± 1.99). Additionally, main effects of Immersion (*F* (1, 56) = 8.63*, p* = 0.005, ${\eta }_{p}^{2}$ = 0.13) and Content (*F* (2, 112) = 56.52*, p* < 0.001, ${\eta }_{p}^{2}$ = 0.50) were found. On average, participants reported greater arousal following exposure to the immersive device (*M*_*HMD*_ = 4.85 ± 2.36) than for the less immersive one (*M*_*Screen*_ = 4.32 ± 2.22). Contrary to our expectations, the Age-Group x Immersion interaction did not reach significance (*F* (1, 56) = 1.10, *p* = 0.30). Regarding the main effect of Video content on self-reported arousal, post-hoc test revealed that social (*M* = 5.48 ± 2.03) and natural (*M* = 5.01 ± 2.19) video contents induced greater arousal than the control content (*M* = 3.26 ± 2.09, *p*_*s*_ < 0.001). A significant Age-Group x Content interaction emerged (*F* (2, 112) = 4.15*, p* = 0.03, ${\eta }_{p}^{2}$ = 0.07), which was driven by older adults reporting higher levels of arousal compared to younger adults for the natural video contents (respectively *M*_*OA*_ = 6.21 ± 2.11,* M*_*YA*_ = 4.16 ± 1.82, *p* < 0.001). However, no age-related differences emerged for the social (*M*_*OA*_ = 6.11 ± 2.20,* M*_*YA*_ = 5.03 ± 1.79, *p* = 0.24) nor the control (*M*_*OA*_ = 3.72 ± 2.40,* M*_*YA*_ = 2.93 ± 1.80, *p* = 1) video content. Furthermore, none of the remaining interactions were significant (all *p*_*s*_ > 0.1).

### Physiological emotional responses

#### Heart rate (HR)

**Figure 3:**
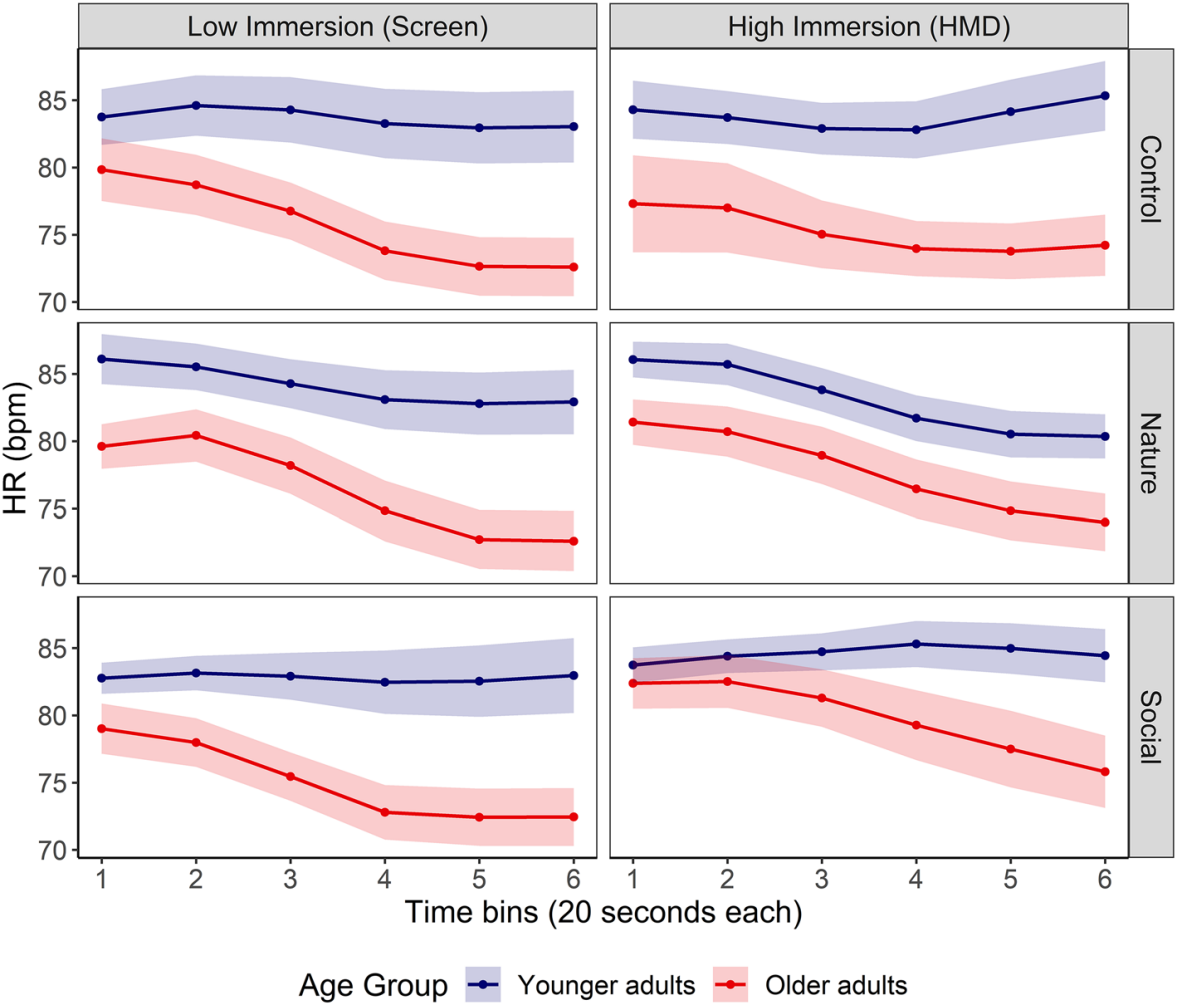
Time-course of younger and older adults’ Heart Rate (HR) while they were watching in both levels of immersion the control, natural and social video contents. Ribbons indicate standard error from the mean.

Growth Curve Analyses (GCA) were conducted to analyze and model the time-course variations in participants’ HR while they were watching the control, natural and social video contents on the low and highly immersive devices (see Fig. 3). Model comparisons revealed that the model including the linear, quadratic and cubic time terms best accounted for the time-course of HR (see Tables S2–S4 in Supplementary Material 2 for model comparison and the detailed results of the selected model).

Overall, both linear (*Estimate* = − 69.59, *SE* = 16.87, *p* < 0.001) and cubic (*Estimate* = 16.83, *SE* = 3.11, *p* < 0.001) polynomials significantly predicted changes in HR, indicating that participants’ HR decreased throughout the video duration.

A significant main effect of Age-Group was observed on the intercept term (*Estimate* = -3.51, *SE* = 1.01, *p* = 0.002), indicating that in general older adults had lower HR (*M* = 76.6 ± 11.2) than younger adults (*M* = 83.7 ± 11.7). Additionally, significant interactions between Age-Group and the linear (*Estimate* = − 43.50, *SE* = 16.87, *p* = 0.01) and cubic terms (*Estimate* = 6.93, *SE* = 3.11, *p* = 0.03) revealed that older adults manifested a greater HR decrease throughout the video presentation relative to younger adults, as illustrated on Fig. 3.

No significant effect of Immersion emerged on the intercept, linear or quadratic term (all *p*_*s*_ > 0.1). Only the interaction between Immersion and the cubic term reached significance (*Estimate* = -4.93, *SE* = 1.54, *p* < 0.001), suggesting that participants manifested initially a greater decrease in HR during the first moments of the exposure to the highly immersive device, followed by a slight rise of HR towards the end compared to the less immersive one.

The main effect of Video content did not emerge on the intercept (*F*_*Content*_ (2, 290) = 0.64, *p* = 0.53). However, significant interactions were found between Content and the linear (*F*_Content*Linear_ (2, 290) = 3.61, *p* = 0.03) and the cubic terms (*F*_Content*Cubic_ (2, 986) = 6.76, *p* = 0.001). Further analyses revealed that natural video contents elicited a greater decrease in HR compared to the control content (linear interaction term, *Estimate* = − 36.52, *SE* = 13.66, *p* = 0.008; cubic interaction term, *Estimate* = 7.57, *SE* = 2.18, *p* < 0.001). Social video contents elicited a less pronounced and more gradual decrease throughout the video presentation compared to the control video content (cubic interaction term, *Estimate* = − 6.03, *SE* = 2.18, *p* = 0.006). Furthermore, a significant interaction was found between Video Content and Level of Immersion on the quadratic term (*F*_Immersion*Content*Quadratic_ (2, 290) = 4.59, *p* = 0.01). As illustrated in Fig. 3**,** social video contents watched on the highly immersive device elicited a quick increase in HR followed by a gradual decrease, whereas they mostly elicited a gradual decrease in participants’ HR in the less immersive condition.

More interestingly, a significant three-way interaction between Age-Group, Level of immersion and video content was evident on the cubic term (*F*_Age-Group*Immersion*Content*Cubic_ (2, 986) = 5.10, *p* = 0.006). To further investigate this interaction, we conducted separate follow-up analyses for low-immersive and high-immersive devices separately (see Tables S5–S8 in Supplementary Material 2 for more details). An Age-Group x Video content interaction on the cubic term was found only for the low immersive device (*F*_Age-Group*Content*Cubic_ (2, 464) = 3.88, *p* = 0.02), whereas the same interaction failed to reach significance for the highly immersive device (*F*_Age-Group*Content*Cubic_ (2, 464) = 2.49, *p* = 0.08). Further analyses pointed out that the Age-Group x Content interaction on the cubic time term reflected age-related differences in HR while participants were exposed to natural contents on the less immersive device. As illustrated in Fig. 3**,** older adults manifested a more pronounced HR decrease compared to younger adults throughout the presentation of natural video contents on the low-immersive device.

#### Skin conductance level (SCL)

**Figure 4:**
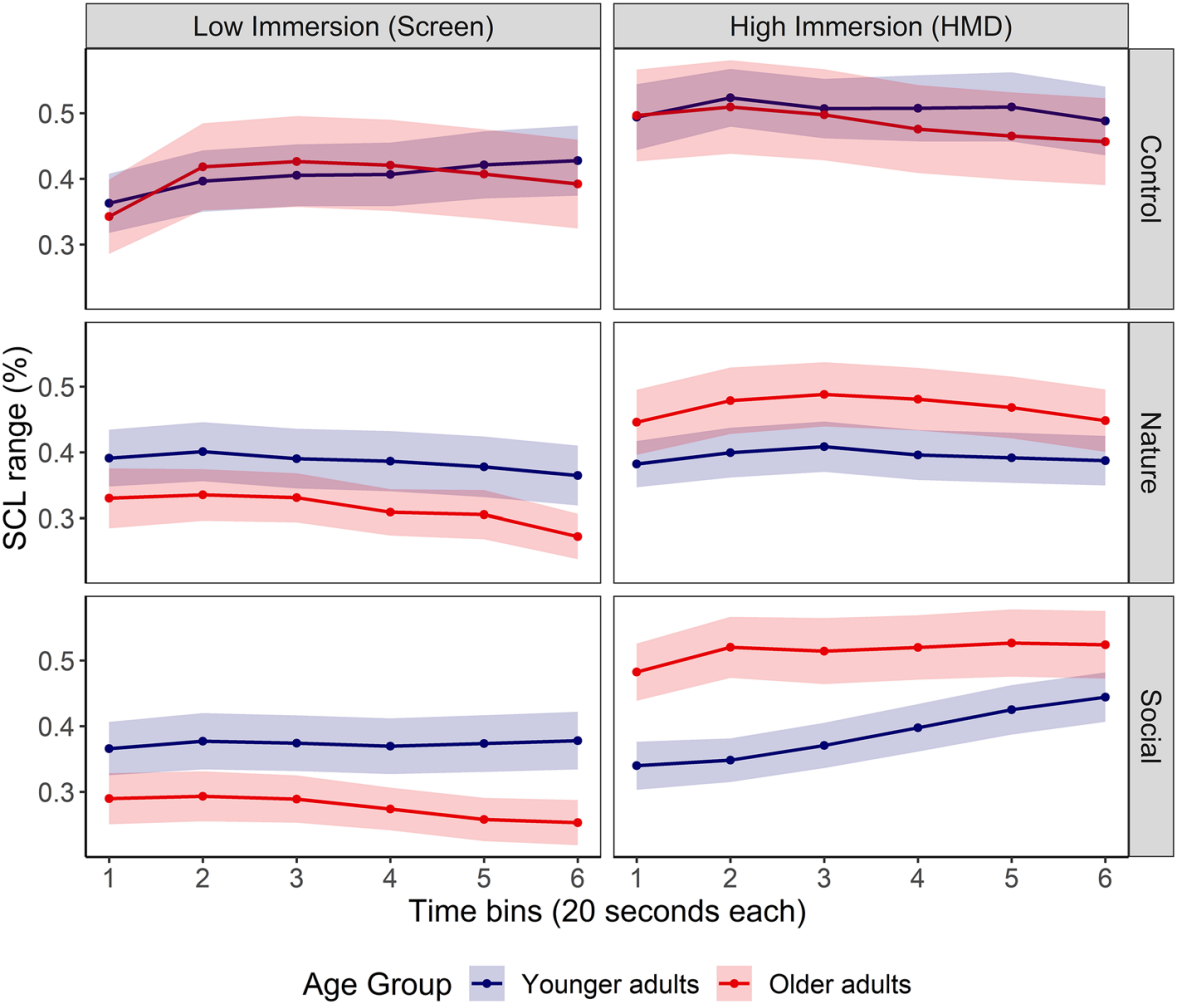
The time course of younger and older adults’ Skin Conductance Level (SCL) range while they were watching in both levels of immersion the control, natural and social video contents. Ribbons indicate standard error from the mean.

GCA were also conducted to model the time course of participants’ SCL while they were watching the control, natural and social video contents on both immersive devices (see Fig. 4). Model comparisons revealed that the GCA model including the linear and quadratic time terms best accounted for the time-course of SCL (see Tables S10–S12 in Supplementary Material 2 for model comparison and the detailed results of the selected model).

Overall, the quadratic polynomial significantly predicted changes in SCL range (*Estimate* = − 0.32, *SE* = 0.08, *p* < 0.001), revealing that participants’ SCL followed a non-linear change with an initial increase followed by a progressive decrease.

The main effect of Age-Group was not evident on the intercept term (*Estimate* = 0.001, *SE* = 0.02*, p* = 0.97). Interestingly, a significant interaction was found between Age-Group and the quadratic time term (*Estimate* = − 0.16*, SE* = 0.08, *p* = 0.04), indicating that younger adults’ SCL increased gradually throughout video presentation, whereas older adults manifested an initial increase in SCL throughout the first half video presentation followed by a decrease.

The main effect of Immersion emerged on the intercept term (*Estimate* = 0.05, *SE* = 0.01*, p* < 0.001), indicating that exposure to the highly immersive device elicited overall greater SCL (*M* = 0.45 ± 0.26) compared to the less immersive one (*M* = 0.36 ± 0.26). Furthermore, the Age-Group x Immersion interaction reached significance on the intercept term (*F*_Age-Group*Immersion_ (1, 290) = 6.03, *p* = 0.01), reflecting that older adults manifested overall lower SCL (*M*_*OA*_ = 0.33 ± 0.23) than younger adults (*M*_*YA*_ = 0.39 ± 0.27) during exposure to the videos on the low-immersive device, whereas the opposite pattern is observed for the highly immersive device (*M*_*OA*_ = 0.49 ± 0.26, *M*_*YA*_ = 0.43 ± 0.24). Neither the main effect of Immersion nor the Age-Group x Immersion interaction reached significance on the linear or the quadratic time terms (all *p*_*s*_ > 0.1).

A tendency for a main effect of video content was found on the intercept term (*F*_*content*_ (2, 290) = 2.76*, p* = 0.06), reflected by an overall lower SCL for both natural (*M* = 0.39 ± 0.23) and social (*M* = 0.39 ± 0.23) contents compared to the control video content (*M* = 0.45 ± 0.30). A marginal interaction was found between video content and the linear term (*F*_Content*Linear_ (2, 290) = 2.97, *p* = 0.05), as well as a significant interaction with the quadratic time term (*F*_Content*Quadratic_ (2, 290) = 4.12, *p* = 0.02). Further analyses revealed that natural video contents elicited a greater decrease in SCL compared to the control content (linear interaction term, *Estimate* = − 0.41, *SE* = 0.19, *p* = 0.03; quadratic interaction term, *Estimate* = 0.37, *SE* = 0.19, *p* = 0.05). Moreover, social video contents elicited a more pronounced increase in SCL compared to the control content (quadratic interaction term, *Estimate* = 0.24, *SE* = 0.08, *p* = 0.005). Additionally, a significant Video Content x Immersion interaction was found on the linear time term (*F*_Content*Immersion*Linear_ (2, 290) = 2.97, *p* < 0.001), which was driven by social video contents (*Estimate* = 0.56, *SE* = 0.19, *p* = 0.003). As illustrated on Fig. 4**,** social video contents elicited overall a steeper increase in SCL when presented on the highly immersive device, but a gradual decrease when presented on the less immersive device.

Lastly a significant three-way interaction of Age-Group, Immersion and Video Content was evident on the quadratic term (*F*_Age-Group*Immersion*Content*Quadratic_ (2, 290) = 3.30, *p* = 0.04). To further investigate this interaction, we conducted separate follow-up analyses for low-immersive and high-immersive devices separately (see Tables S13, S14 in Supplementary Material 2 for more details). Although Fig. 4 seemed to suggest that natural and social video contents elicited opposite age-related changes in SCL depending on the level of immersion, this observation failed to emerge in follow-up analyses (all *p*_*s*_ > 0.01).

### Correlations between self-reported and physiological emotional responses

On an exploratory basis, Pearson’s *r* correlation coefficients were computed between participants' self-reported and averaged physiological responses. None of the correlations were significant (all *p*_*s*_ > 0.1). Furthermore, no significant correlations emerged when examining younger and older adults' emotional responses separately (all *p*_*s*_ > 0.1).

### Sense of presence

Spatial and Social Sense of Presence (SoP) were analyzed with ANOVAs including Age-Group (Younger *vs* Older adults) as between-subject factor, Level of Immersion (low *vs* high) and Video Contents (Control *vs* Natural *vs* Social) as within-subject factors.

#### Spatial sense of presence

Results showed a significant Age-Group effect (*F* (1, 56) = 7.22*, p* = 0.01, ${\eta }_{p}^{2}$ = 0.11), indicating that older participants reported greater spatial SoP (*M* = 3.40 ± 1.16) compared to younger adults (*M* = 2.85 ± 1.08). A main effect of Immersion was observed (*F* (1, 56) = 36.17*, p* < 0.001, ${\eta }_{p}^{2}$ = 0.39), indicating that exposure to the highly immersive device induced greater spatial SoP (*M* = 2.73 ± 1.15) than the less immersive one (*M* = 3.43 ± 1.02). This finding was further modulated by an Age-Group x Immersion interaction (*F* (1, 56) = 12.95*, p* < 0.001, ${\eta }_{p}^{2}$ = 0.20) revealing that older adults reported higher spatial SoP (*M*_*OA*_ = 3.28 ± 1.20) for the low-immersive device compared to younger adults (*M*_*YA*_ = 2.34 ± 0.95, *p* < 0.001), while no age-related differences emerged following exposure to the highly immersive device (*M*_*OA*_ = 3.52 ± 1.11, *M*_*YA*_ = 3.36 ± 0.96, *p* = 0.49). Additionally, a significant a main effect of Content emerged (*F* (2, 112) = 51.93*, p* < 0.001, ${\eta }_{p}^{2}$ = 0.48), as participants reported higher levels of spatial SoP for natural (*M* = 3.32 ± 1.09) and social video contents (*M* = 3.33 ± 1.10) compared to the control one (*M* = 2.58 ± 1.09, *p*_*s*_ < 0.001). A tendency for an Age-Group x Content interaction was observed (*F* (2, 112) = 3.29*, p* = 0.05, ${\eta }_{p}^{2}$ = 0.06), indicating that older adults reported greater spatial SoP than younger adults for natural video contents (*M*_*OA*_ = 3.77 ± 1.02, *M*_*YA*_ = 3.00 ± 1.03, *p* = 0.02), while no age-related differences emerged for social (*M*_*OA*_ = 3.66 ± 1.06, *M*_*YA*_ = 3.10 ± 1.08,* p* = 0.25) nor the control video contents (*M*_*OA*_ = 2.77 ± 1.15, *M*_*YA*_ = 2.45 ± 1.03,* p* = 1.00). The remaining interactions failed to reach significance (all *p*_*s*_ > 0.1).

#### Social sense of presence

Results showed a significant Age-Group effect (*F* (1, 56) = 23.44*, p* < 0.001, ${\eta }_{p}^{2}$ = 0.30), indicating that older participants reported greater social SoP (*M* = 4.85 ± 1.81) compared to younger adults (*M* = 3.85 ± 1.53). Immersion had a significant effect on social SoP ratings (*F* (1, 56) = 16.81*, p* < 0.001, ${\eta }_{p}^{2}$ = 0.23), indicating that higher levels of immersion induced greater social SoP (*M* = 4.52 ± 1.65) compared to lower levels of immersion (*M* = 4.01 ± 1.76). Additionally, a significant a main effect of Content emerged (*F* (2, 112) = 166.54*, p* < 0.001, ${\eta }_{p}^{2}$ = 0.75), indicating that social contents induced highest scores of social SoP (*M* = 5.33 ± 1.12), followed by natural contents (*M* = 4.76 ± 1.35) and lastly control content (*M* = 2.71 ± 1.42, all *p*_*s*_ < 0.01). A significant Age-Group x Immersion interaction was observed (*F* (1, 56) = 7.54*, p* = 0.009, ${\eta }_{p}^{2}$ = 0.12). However, post-hoc analyses only further confirmed the main effect of age-group on social SoP, with older adults reporting higher social SoP ratings than younger adults for both the less immersive (*M*_*OA*_ = 4.77 ± 1.80, *M*_*YA*_ = 3.48 ± 1.52, *p* < 0.001) and the highly immersive device (*M*_*OA*_ = 4.93 ± 1.83, *M*_*YA*_ = 4.23 ± 1.45, *p* = 0.02). The Age-Group x Content interaction was also significant (*F* (2, 112) = 6.48*, p* = 0.002, ${\eta }_{p}^{2}$ = 0.10), revealing that older adults reported greater social SoP than younger adults for natural video contents (*M*_*OA*_ = 5.72 ± 1.15, *M*_*YA*_ = 4.09 ± 1.05, *p* < 0.001), while no age-related differences emerged for the social (*M*_*OA*_ = 5.69 ± 1.15, *M*_*YA*_ = 5.07 ± 1.03, *p* = 0.36) nor the control video contents (*M*_*OA*_ = 3.14 ± 1.69, *M*_*YA*_ = 2.40 ± 1.10, *p* = 0.10). The remaining interactions failed to reach significance (all *p*_*s*_ > 0.1).

## Discussion

The aim of the present study was to investigate age-related differences in self-reported and physiological emotional responses elicited by immersion in virtual environments which were designed to induce positive emotions by featuring either natural or social content.

Regarding subjective emotional responses, our findings revealed age-related differences solely on arousal but not valence ratings. The lack of age-related differences in valence ratings confirms that the presently employed material (i.e., immersion in natural and social video contents) elicited successfully positive emotions in both age-groups. This is further supported by the effect of immersion level and video content on participants' valence ratings. Indeed, higher levels of immersion enhanced positive emotions across all participants. Additionally, natural and social video content elicited significantly more positive emotions in both age-groups than the neutral content. Concerning arousal ratings, in line with previous studies^18,25^, older adults provided overall more extreme arousal ratings than younger adults. This age-related tendency was modulated by the content of the videos, since older adults reported significantly higher levels of arousal than younger adults for the natural (i.e., non-social) video content, while no age-related difference emerged in the arousal ratings reported following the viewing of the control (i.e., neutral) nor the social video content. As reported previously^24,25^, the lack of age-related differences in the control condition suggests that our results highlight an age-related increase in self-reported arousal in response to affective information, rather than a response bias in older adults. Lastly, exposure to the highly immersive device elicited in all participants higher levels of arousal compared to the less immersive device. Taken together, these findings shed light on the superior benefits of highly immersive devices to elicit positive emotions in older adults in contrast to earlier studies^50,53^ and demonstrate the suitability of natural and social contents to elicit positive emotions in young and older adults.

Our findings regarding Heart Rate (HR) revealed mostly age-related difference in the time course of participants’ physiological responses to the video contents. In line with results usually reported in the litterature^26,83^, older adults exhibited overall lower HR than younger adults. Superseding this general effect of age on HR, the time-series analyses revealed that older adults manifested a more pronounced HR decrease than younger adults while viewing the videos. Contrary to our expectation, this age-related difference was not modulated by the level of immersion. Nevertheless, older adults manifested the greatest HR decrease over time during exposure to natural video contents, especially on the low immersive device. This finding might reflect a heightened state of relaxation in older adults compared to younger adults when exposed to natural contents, as suggested by previous studies^46,49,67^. In contrast, social contents, especially when presented on the highly immersive device, elicited a gradual increase in HR in all participants, which could be assimilated to the elicitation of a more arousing emotion such as joy or happiness^84^. Given that HR is influenced by both the sympathetic and parasympathetic nervous systems^85,86^, employing finer indices like Heart Rate Variability may be useful to gain a thorough understanding of content's influence on emotional responses^86–88^.

The time-course analyses of Skin Conductance Level (SCL) complemented previously discussed results and highlighted the influence of immersion on physiological responses. As for HR, age-related differences emerged in the time-course of SCL: while younger adults manifested a gradual increase in SCL, older adults manifested more transient SCL over time, providing additional support regarding the relevance of time-series analyses for bringing better understanding of age-related differences in emotional responses. Our findings also supported the general effect of content on physiological responses: overall, natural (i.e., nonsocial) contents tended to elicit a decrease in SCL overtime, whereas social contents elicited rather an increase of SCL. Moreover, highly immersive devices elicited overall a greater SCL compared to less immersive devices, in accordance with previous studies indicating an increase of arousal when viewing stimuli on a highly immersive device^52,89^. Surprisingly, however, older adults manifested lower SCL than younger counterparts on the less immersive device, whereas the inverse pattern was observable for the less immersive device. As electrodermal activity is known to change according to stimulus intensity but also because of a rise in attention or stimulus novelty^90^, it is plausible that the results observed in older adults while being exposed to the highly immersive device reflects the involvement of another mechanism going beyond the sole level of physiological arousal in which we were interested. Hence, the reported results regarding SCL could enlighten while older adults manifested a deeper relaxation for natural contents while viewing them on the less immersive device. Overall, our findings support both levels of immersion for eliciting positive emotions in older adults and highlight the importance of content in immersive experiences.

Taken together, our observations may suggest age-related differences in appraisal of ones’ peripheral responses. In older adults, a pronounced physiological relaxation seems to be appraised afterwards as an intense emotional experience on a subjective level. In contrast, younger adults seem to appraise their subjective responses according to the magnitude of their peripheral responses. While the mechanisms underlying age-related difference in appraisal require further investigation, they may be attributed in our opinion to the shift in motivational goals that occurs in aging in line with the SST^14^. As older adults prioritize their well-being, they may seek to potentialize benefits from positive experiences. Conversely, younger adults may be more inclined to seek out novel experiences and social interactions, thus manifesting a greater response and interest towards social scenarios. Nevertheless, our interpretations must be considered carefully as no clear association emerged between participants' self-reported and physiological emotional responses. This lack of emotional coherence is not uncommon^24,70,91^, and can be interpreted in the light of dual-process perspective^91^ according to which physiological responses reflect "automatic" reactions, as they are often measured continuously during stimuli exposure, while self-reported data reflects "reflective" responses modulated by top-down processes as their assessment usually takes place after stimuli exposure. Integrating continuous self-reported measures during stimuli exposure could provide valuable insights, though implementing them in VR presents challenges^92,93^. An alternative interpretation of our results may stem from age-related differences in the interpretation of self-reported questionnaires, since some research suggests that older people experience more complex and mixed subjective emotional experiences than young adults^54^. In sum, our results highlight the complex nature of age-related differences in emotional responses to positive emotion and prompt further exploration.

Our study offers additional practical insights into the use of VR for enhancing emotional well-being of young and older adults. In the first instance, it confirms the benefits of exposure to virtual nature in younger^64–66,94,95^ and older^46,49,67^ users, while also highlighting the potential of social video content for enhancing their emotional well-being. Interestingly, older adults reported heightened spatial and social sense of presence compared to younger adults, even when using the less immersive device, challenging the assumption that high immersion is needed for emotional induction^51,57^. Hence, further studies are needed to examine which aspects of VR devices (e.g., physical comfort^55^) might have an impact on older adults’ sense of presence. Taken together, our observations highlight the need for user-centered design in the development of VR applications^13,96,97^, which requires additional examination of the benefits of VR beyond our highly educated and technologically competent sample to ensure its efficacy and safety for all users, including the most vulnerable ones.

It is important to acknowledge the limitations of the present study, despite addressing several issues concerning age-related differences in emotional responses to positive emotion induction with VR. A primary constraint was the absence of established guidelines for the optimal VR exposure duration to effectively induce positive emotions. While our study demonstrated that a two-minute VR exposure can elicit significant emotional responses, this is shorter than the immersion duration often reported in previous VR-based research^46,49,66,87,94,98,99^. These methodological divergences raise questions about the minimal effective exposure time for eliciting positive emotions across age groups and observing changes in physiological responses. Nevertheless, the present results suggest that even short durations can be impactful to elicit positive emotions and enhance users’ well-being. Another limitation lies in the difference of video format between our control and experimental stimuli. Only the control video content was computer-generated because it enables a strict control of the emotional neutrality of said stimuli^100^ and adhering to existing guidelines for that matter^99^. Although it seems there are little-to-no differences in self-reported and/or physiological responses elicited by these two formats^95,101^, current literature offers limited insights on this aspect.

In conclusion, our study has significant theoretical and practical implications. Theoretically, the use of more ecologically valid and engaging VR content has enhanced our understanding of older adults' emotional responses in real-life situations. More specifically, our results highlighted age-related differences in the time course of participants' physiological responses, as older adults manifested a more pronounced decline of their physiological responses over time, while younger adults manifested more sustained changes in physiological indices over time and according to the content of the stimuli. Moreover, these age-related differences in physiological responses seem to be appraised differently as a function of age. On a practical ground, our study presents compelling evidence of VR’s efficacy in eliciting positive emotions, as VR offers an almost instant sense of escape and well-being for both younger and older individuals. Moreover, our findings emphasize the importance of considering the content of the virtual environment to improve users’ emotional well-being. In addition, our results provide new avenues for exploring the technical aspects and cognitive mechanisms that contribute to emotion induction with VR in the elderly, as these mechanisms appear to differ from those observed in younger adults. Overall, our study offers promising insight for future research aiming to investigate age-related differences in emotion processing and enhancing emotional well-being in older adults with VR.

### Supplementary Information

Supplementary Tables.

Supplementary Information.

## Data Availability

The raw data supporting the conclusions of this article will be made available on request to the corresponding author, without undue reservation.
