# Supplementary material for: Age-related differences in subjective and physiological emotion evoked by immersion in natural and social virtual environments
Source: Sci Rep. 2024 Jul 3;14:15320. doi: 10.1038/s41598-024-66119-5 (PMC11222553; doi:10.1038/s41598-024-66119-5)
Supplement: Supplementary file 1 — Supplementary Tables. [file 41598_2024_66119_MOESM1_ESM.doc]

**Supplementary material 1**

**Supplementary Table 1.** Summary of younger adults self-reported positive and negative affect.

**Supplementary Table 2.** Summary of older adults self-reported positive and negative affect.

**Supplementary Table 3.** Summary of the Group x Immersion x Content ANOVA on self-reported positive emotional responses.

**Supplementary Table 4.** Summary of the Group x Immersion x Content ANOVA on self-reported negative emotional responses.

**Significance levels**: p < 0.07: “ ~ ”, p < 0.05: “ * ”, p < 0.01: “ ** ”, p < 0.001: “ *** ”, Non-significant result: “NS”

**Abbreviation:** HMD - Head Mounted Display; YA - Young adults, OA - Older adults.

**Supplementary Table 1.**

|  | | *Video contents* | | |
| --- | --- | --- | --- | --- |
| Immersion | Affects /7 | Control *(M ± SD)* | Nature *(M ± SD)* | Social *(M ± SD)* |
| **Low (Screen)** | | | | |
|  | **Average Positive affect** | 4.09 ± 1.26 | 4.46 ± 0.79 | 4.86 ± 0.80 |
|  | Excitement | 4.50 ± 1.21 | 3.41 ± 1.24 | 4.25 ± 1.07 |
|  | Joy | 4.94 ± 0.95 | 4.68 ± 0.89 | 5.09 ± 0.97 |
|  | Relaxation | 4.62 ± 1.35 | 5.18 ± 1.03 | 4.94 ± 0.94 |
|  | Interest | 5.79 ± 0.59 | 4.56 ± 1.20 | 5.13 ± 0.97 |
|  | **Average Negative affect** | 1.81 ± 0.68 | 1.65 ± 0.52 | 1.48 ± 0.41 |
|  | Anxiety | 2.38 ± 1.07 | 1.72 ± 1.05 | 1.72 ± 0.91 |
|  | Anger | 1.21 ± 0.41 | 1.16 ± 0.36 | 1.13 ± 0.31 |
|  | Sadness | 1.62 ± 0.75 | 1.38 ± 0.62 | 1.24 ± 0.41 |
|  | Boredom | 1.62 ± 1.16 | 2.34 ± 1.27 | 1.81 ± 0.95 |
| **High (Head-Mounted Display)** | | | | |
|  | **Average Positive Affect** | 4.47 ± 0.96 | 4.91 ± 0.87 | 5.14 ± 0.92 |
|  | Excitement | 3.97 ± 1.42 | 4.13 ± 1.17 | 4.74 ± 1.31 |
|  | Joy | 4.41 ± 1.16 | 5.18 ± 0.85 | 5.41 ± 1.01 |
|  | Relaxation | 4.56 ± 1.28 | 5.16 ± 1.32 | 4.91 ± 1.14 |
|  | Interest | 4.94 ± 1.39 | 5.15 ± 0.94 | 5.50 ± 0.92 |
|  | **Average Negative Affect** | 1.63 ± 0.60 | 1.45 ± 0.45 | 1.42 ± 0.42 |
|  | Anxiety | 2.12 ± 1.17 | 4.02 ± 1.24 | 4.75 ± 1.37 |
|  | Anger | 1.26 ± 0.45 | 5.23 ± .80 | 5.58 ± .92 |
|  | Sadness | 1.32 ± 0.64 | 5.25 ± .97 | 5.04 ± .97 |
|  | Boredom | 1.82 ± 1.22 | 5.23 ± .94 | 5.56 ± .90 |

**Supplementary Table 2.**

|  | | *Video contents* | | |
| --- | --- | --- | --- | --- |
| Immersion | Affects /7 | Control *(M ± SD)* | Nature *(M ± SD)* | Social *(M ± SD)* |
| **Low (Screen)** | | | | |
|  | **Average Positive affect** | 3.27 ± 1.00 | 4.72 ± 1.11 | 4.64 ± 1.13 |
|  | Excitement | 3.12 ± 1.60 | 3.31 ± 1.67 | 3.38 ± 1.52 |
|  | Joy | 4.33 ± 1.37 | 4.58 ± 1.40 | 4.83 ± 1.43 |
|  | Relaxation | 4.83 ± 1.97 | 5.73 ± 1.26 | 5.48 ± 1.36 |
|  | Interest | 5.96 ± 1.04 | 5.25 ± 1.22 | 4.96 ± 1.28 |
|  | **Average Negative affect** | 1.44 ± 0.52 | 1.31 ± 0.67 | 1.22 ± 0.36 |
|  | Anxiety | 1.71 ± 1.00 | 1.29 ± 0.72 | 1.15 ± 0.31 |
|  | Anger | 1.50 ± 1.18 | 1.21 ± 0.44 | 1.27 ± 0.51 |
|  | Sadness | 1.42 ± 0.72 | 1.33 ± 0.87 | 1.21 ± 0.41 |
|  | Boredom | 1.54 ± 1.14 | 1.40 ± 0.91 | 1.25 ± 0.55 |
| **High (Head-Mounted Display)** | | | | |
|  | **Average Positive Affect** | 3.94 ± 1.28 | 5.11 ± 1.10 | 4.99 ± 1.26 |
|  | Excitement | 2.58 ± 1.53 | 3.90 ± 1.96 | 3.83 ± 1.87 |
|  | Joy | 3.62 ± 1.56 | 4.96 ± 1.28 | 5.00 ± 1.53 |
|  | Relaxation | 4.71 ± 1.99 | 5.75 ± 1.06 | 5.40 ± 1.25 |
|  | Interest | 4.83 ± 1.74 | 5.81 ± 1.03 | 5.52 ± 1.36 |
|  | **Average Negative Affect** | 1.31 ± 0.60 | 1.26 ± 0.44 | 1.33 ± 0.55 |
|  | Anxiety | 1.38 ± 0.77 | 1.25 ± 0.66 | 1.21 ± 0.49 |
|  | Anger | 1.29 ± 0.69 | 1.23 ± 0.49 | 1.52 ± 0.91 |
|  | Sadness | 1.21 ± 0.66 | 1.21 ± 0.57 | 1.23 ± 0.51 |
|  | Boredom | 1.38 ± 0.65 | 1.31 ± 0.64 | 1.42 ± 0.65 |

**Supplementary Table 3.**

| **Effect** | **Test** | **Comparisons** | **Results** | **Significance** |
| --- | --- | --- | --- | --- |
| **Age-Group** |  |  | F(1,56) = 1.07, p = 0.31 | NS |
| **Immersion** |  |  | F(1,56) = 24.04, p < 0.001 | *** |
| **Content** |  |  | F(2,112) = 38.62, p < 0.001 | *** |
|  | Post-hoc | Control – Nature | p < 0.001 | *** |
|  |  | Control – Social | p < 0.001 | *** |
|  |  | Nature – Social | p = 1.00 | NS |
| **Age-Group x Immersion** |  |  | F(1, 56) =0.30, p = 0.59 | NS |
| **Age-Group x Content** |  |  | F(2, 112) = 7.13, p = 0.001 | *** |
|  | Post-hoc | YA Control – OA Control | p = 0.10 | NS |
|  |  | YA Control – YA Nature | p = 0.16 | NS |
|  |  | YA Control – OA Nature | p = 0.16 | NS |
|  |  | YA Control – YA Social | p < 0.001 | *** |
|  |  | YA Control – OA Social | p = 0.45 | NS |
|  |  | OA Control – YA Nature | p < 0.001 | *** |
|  |  | OA Control – OA Nature | p < 0.001 | *** |
|  |  | OA Control – YA Social | p < 0.001 | *** |
|  |  | OA Control – OA Social | p < 0.001 | *** |
|  |  | YA Nature – OA Nature | p = 1.00 | NS |
|  |  | YA Nature – YA Social | p = 0.63 | NS |
|  |  | YA Nature – OA Social | p = 1.00 | NS |
|  |  | OA Nature – YA Social | p = 1.00 | NS |
|  |  | OA Nature – OA Social | p = 1.00 | NS |
|  |  | OA Social – YA Social | p = 1.00 | NS |
| **Immersion x Content** |  |  | F(2,112) = 0.80, p = 0.45 | NS |
| **Age-Group x Immersion x Content** |  |  | F(2,112) = 0.59, p = 0.56 | NS |

**Supplementary Table 4.**

| **Effect** | **Test** | **Comparisons** | **Results** | **Significance** |
| --- | --- | --- | --- | --- |
| **Age-Group** |  |  | F(1,56) = 6.69, p = 0.01 | ** |
| **Immersion** |  |  | F(1,56) = 4.62, p = 0.04 | * |
| **Content** |  |  | F(2,112) = 4.47, p = 0.02 | ** |
|  | Post-hoc | Control – Nature | p = 0.12 | NS |
|  |  | Control – Social | p = 0.01 | * |
|  |  | Nature – Social | p = 1.00 | NS |
| **Age-Group x Immersion** |  |  | F(1, 56) = 0.08, p = 0.12 | NS |
| **Age-Group x Content** |  |  | F(2, 112) = 0.89, p = 0.41 | NS |
| **Immersion x Content** |  |  | F(2,112) = 2.52, p = 0.10 | NS |
| **Age-Group x Immersion x Content** |  |  | F(2,112) = 0.26, p = 0.73 | NS |
