# Supplementary material for: Age-related differences in subjective and physiological emotion evoked by immersion in natural and social virtual environments
Source: Sci Rep. 2024 Jul 3;14:15320. doi: 10.1038/s41598-024-66119-5 (PMC11222553; doi:10.1038/s41598-024-66119-5)
Supplement: Supplementary file 2 — Supplementary Information. [file 41598_2024_66119_MOESM2_ESM.pdf]

Supplementary Material 2 - Growth Curve Analyses

Contents

1 GCA on Heart Rate (HR) **2**

1.1 Dataframe manipulation 2

1.1.1 Pivot dataframe from wide to long format 2

1.1.2 Define factors and their levels 2

1.2 Creation of the polynomials 2

1.3 Creation of the contrasts (Sum coding) 2

1.4 **Supplementary Table 1.** Descriptive statistics for HR Data 3

1.5 **Supplementary Figure 1.** Time-course of younger and older adults' Heart Rate 4

1.6 GCA on HR - Results 5

1.7 **Supplementary Table 2.** Model comparison 6

1.8 **Supplementary Table 3.** Results of the selected model 6

1.9 **Supplementary Table 4.** Estimates for significant effects and interactions 7

1.9.1 GCA on HR for Screen 10

1.9.2 GCA on HR for VR 13

2 GCA on Skin Conductance Level (SCL) **15**

2.1 Dataframe manipulation 15

2.1.1 Pivot dataframe from wide to long format 16

2.1.2 Define factors and their levels 16

2.2 Creation of the polynomials 17

2.3 Creation of the contrasts (Sum coding) 17

2.4 **Supplementary Table 9.** Descriptive statistics for SCL data 17

2.5 **Supplementary Figure 2.** Plot of SCL time course 19

2.6 GCA on SCL - Results 20

2.7 **Supplementary Table 10.** Models Comparisons 21

2.8 **Supplementary Table 11.** Results of the selected model 21

2.9 **Supplementary Table 12.** Estimates for significant effects and interactions 22

2.9.1 GCA on SCL for SC 25

2.9.2 GCA on SCL for VR 25

Data input

```
HR <- read.csv2(here::here("data", "GCA_HR.csv"), fileEncoding="UTF-8-BOM")
SCL <- read.csv2(here::here("data", "GCA_SCL.csv"), fileEncoding="UTF-8-BOM")
```

1 GCA on Heart Rate (HR)

1.1 Dataframe manipulation

```
HR$Group <- as.factor(HR$Group)
HR$Group <- revalue(HR$Group, c("JA"= "Younger adults", "PA" = "Older adults"))
HR <- HR %>%
  rename(
    "Age Group" = Group
  )
```

1.1.1 Pivot dataframe from wide to long format

```
HR_long <- HR %>%
  pivot_longer(names_to = c("Immersion","Content","bin"),
               names_sep = "_",
               -c(Sujet,  "Age Group"))
```

1.1.2 Define factors and their levels

```
HR_long$bin <- as.numeric(HR_long$bin)
HR_long$Immersion <- as.factor(HR_long$Immersion)
HR_long$Immersion <- revalue(HR_long$Immersion, c("EC"="Low Immersion (Screen)", "VR"= "High Immersion (HM
HR_long$Content <- factor(HR_long$Content)
HR_long$Content <- revalue(HR_long$Content, c("Tuto"="Control", "NS"= "Nature", "S" = "Social"))
HR_long$Content <- factor(HR_long$Content, levels = c("Control", "Nature", "Social"))
HR_long$Sujet <- as.factor(HR_long$Sujet)
```

1.2 Creation of the polynomials

```
polynomials <- poly(HR_long$bin, degree = 4)
HR_long$ot1 <- polynomials[,1]
HR_long$ot2 <- polynomials[,2]
HR_long$ot3 <- polynomials[,3]
HR_long$ot4 <- polynomials[,4]
```

1.3 Creation of the contrasts (Sum coding)

```
HR_long$ContentS <- contr_code_sum(HR_long$Content, omit = 1) # Keeps the control content as -1 for both c
HR_long$GroupS <- ifelse(HR_long$`Age Group` == "Older adults", 1, -1)
HR_long$ImmersionS <- ifelse(HR_long$Immersion == "High Immersion (HMD)", 1, -1)
```

1.4 Supplementary Table 1. Descriptive statistics for HR Data

```
mse_HR <- ddply(HR_long, c("`Age Group`", "Immersion", "Content", "bin"), summarise,
               N    = length(unique(Sujet)),
               mean = mean(value, na.rm= TRUE),
               sd   = sd(value, na.rm = TRUE),
               se   = sd / sqrt(N))

mse_HR
```

```
##          Age Group              Immersion Content bin  N mean    sd   se
## 1  Younger adults Low Immersion (Screen) Control   1 34 83.8 12.05 2.07
## 2  Younger adults Low Immersion (Screen) Control   2 34 84.6 13.06 2.24
## 3  Younger adults Low Immersion (Screen) Control   3 34 84.3 14.17 2.43
## 4  Younger adults Low Immersion (Screen) Control   4 34 83.3 15.02 2.58
## 5  Younger adults Low Immersion (Screen) Control   5 34 82.9 15.40 2.64
## 6  Younger adults Low Immersion (Screen) Control   6 34 83.0 15.54 2.66
## 7  Younger adults Low Immersion (Screen)  Nature   1 34 86.1 10.82 1.86
## 8  Younger adults Low Immersion (Screen)  Nature   2 34 85.5 10.04 1.72
## 9  Younger adults Low Immersion (Screen)  Nature   3 34 84.3 10.59 1.82
## 10 Younger adults Low Immersion (Screen)  Nature   4 34 83.1 12.73 2.18
## 11 Younger adults Low Immersion (Screen)  Nature   5 34 82.8 13.50 2.32
## 12 Younger adults Low Immersion (Screen)  Nature   6 34 82.9 13.93 2.39
## 13 Younger adults Low Immersion (Screen)  Social   1 34 82.8  6.72 1.15
## 14 Younger adults Low Immersion (Screen)  Social   2 34 83.1  7.48 1.28
## 15 Younger adults Low Immersion (Screen)  Social   3 34 82.9 10.15 1.74
## 16 Younger adults Low Immersion (Screen)  Social   4 34 82.5 13.72 2.35
## 17 Younger adults Low Immersion (Screen)  Social   5 34 82.5 15.48 2.66
## 18 Younger adults Low Immersion (Screen)  Social   6 34 83.0 16.21 2.78
## 19 Younger adults   High Immersion (HMD) Control   1 34 84.3 12.57 2.16
## 20 Younger adults   High Immersion (HMD) Control   2 34 83.7 11.44 1.96
## 21 Younger adults   High Immersion (HMD) Control   3 34 82.9 11.18 1.92
## 22 Younger adults   High Immersion (HMD) Control   4 34 82.8 12.33 2.11
## 23 Younger adults   High Immersion (HMD) Control   5 34 84.1 13.93 2.39
## 24 Younger adults   High Immersion (HMD) Control   6 34 85.3 15.07 2.59
## 25 Younger adults   High Immersion (HMD)  Nature   1 34 86.1  7.72 1.32
## 26 Younger adults   High Immersion (HMD)  Nature   2 34 85.7  8.97 1.54
## 27 Younger adults   High Immersion (HMD)  Nature   3 34 83.8  9.40 1.61
## 28 Younger adults   High Immersion (HMD)  Nature   4 34 81.7  9.90 1.70
## 29 Younger adults   High Immersion (HMD)  Nature   5 34 80.5 10.07 1.73
## 30 Younger adults   High Immersion (HMD)  Nature   6 34 80.4  9.54 1.64
## 31 Younger adults   High Immersion (HMD)  Social   1 34 83.7  7.68 1.32
## 32 Younger adults   High Immersion (HMD)  Social   2 34 84.4  7.27 1.25
## 33 Younger adults   High Immersion (HMD)  Social   3 34 84.7  8.01 1.37
## 34 Younger adults   High Immersion (HMD)  Social   4 34 85.3 10.01 1.72
## 35 Younger adults   High Immersion (HMD)  Social   5 34 85.0 10.95 1.88
## 36 Younger adults   High Immersion (HMD)  Social   6 34 84.4 11.50 1.97
## 37   Older adults Low Immersion (Screen) Control   1 24 79.8 11.42 2.33
## 38   Older adults Low Immersion (Screen) Control   2 24 78.7 10.97 2.24
## 39   Older adults Low Immersion (Screen) Control   3 24 76.8 10.36 2.12
## 40   Older adults Low Immersion (Screen) Control   4 24 73.8 10.60 2.16
## 41   Older adults Low Immersion (Screen) Control   5 24 72.7 10.63 2.17
## 42   Older adults Low Immersion (Screen) Control   6 24 72.6 10.59 2.16
## 43   Older adults Low Immersion (Screen)  Nature   1 24 79.6  8.11 1.65
## 44   Older adults Low Immersion (Screen)  Nature   2 24 80.4  9.52 1.94
```

```
## 45   Older adults Low Immersion (Screen)  Nature   3 24 78.2 10.24 2.09
## 46   Older adults Low Immersion (Screen)  Nature   4 24 74.8 10.99 2.24
## 47   Older adults Low Immersion (Screen)  Nature   5 24 72.7 10.69 2.18
## 48   Older adults Low Immersion (Screen)  Nature   6 24 72.6 10.88 2.22
## 49   Older adults Low Immersion (Screen)  Social   1 24 79.0  9.12 1.86
## 50   Older adults Low Immersion (Screen)  Social   2 24 78.0  8.85 1.81
## 51   Older adults Low Immersion (Screen)  Social   3 24 75.5  8.81 1.80
## 52   Older adults Low Immersion (Screen)  Social   4 24 72.8  9.92 2.03
## 53   Older adults Low Immersion (Screen)  Social   5 24 72.4 10.44 2.13
## 54   Older adults Low Immersion (Screen)  Social   6 24 72.4 10.55 2.15
## 55   Older adults   High Immersion (HMD) Control   1 24 77.3 17.64 3.60
## 56   Older adults   High Immersion (HMD) Control   2 24 77.0 16.25 3.32
## 57   Older adults   High Immersion (HMD) Control   3 24 75.0 12.32 2.52
## 58   Older adults   High Immersion (HMD) Control   4 24 74.0 10.04 2.05
## 59   Older adults   High Immersion (HMD) Control   5 24 73.8 10.11 2.06
## 60   Older adults   High Immersion (HMD) Control   6 24 74.2 11.11 2.27
## 61   Older adults   High Immersion (HMD)  Nature   1 24 81.4  8.25 1.68
## 62   Older adults   High Immersion (HMD)  Nature   2 24 80.7  9.13 1.86
## 63   Older adults   High Immersion (HMD)  Nature   3 24 78.9 10.43 2.13
## 64   Older adults   High Immersion (HMD)  Nature   4 24 76.5 10.67 2.18
## 65   Older adults   High Immersion (HMD)  Nature   5 24 74.8 10.64 2.17
## 66   Older adults   High Immersion (HMD)  Nature   6 24 74.0 10.42 2.13
## 67   Older adults   High Immersion (HMD)  Social   1 24 82.4  9.19 1.87
## 68   Older adults   High Immersion (HMD)  Social   2 24 82.5  9.58 1.96
## 69   Older adults   High Immersion (HMD)  Social   3 24 81.3 10.44 2.13
## 70   Older adults   High Immersion (HMD)  Social   4 24 79.3 12.69 2.59
## 71   Older adults   High Immersion (HMD)  Social   5 24 77.5 13.94 2.84
## 72   Older adults   High Immersion (HMD)  Social   6 24 75.8 13.16 2.69
```

1.5 Supplementary Figure 1. Time-course of younger and older adults' Heart Rate

```
ghr<- ggplot(mse_HR, aes (x = bin, y=mean, fill =`Age Group`))+
  geom_line(lwd=0.75, aes(color=`Age Group`))+
    geom_point(size = 1.3,aes(color=`Age Group`))+
  geom_ribbon(aes(ymin = mean-se, ymax = mean+se, fill=`Age Group`), alpha = .2) +
   facet_grid(Content~Immersion)+
   ylab(" HR (bpm)")+
   theme_bw() +
  theme(legend.position = "bottom",
              axis.title = element_text(size=14),
        axis.text = element_text(size=12),
        legend.text = element_text(size = 12),
        legend.title = element_text(size = 14),
        strip.text = element_text(size=12.5),
        panel.grid.major = element_blank(), panel.grid.minor = element_blank(),
panel.background = element_blank(), axis.line = element_line(colour = "black"))+
  scale_color_manual(values = c("#000271", "#E70007"))+
  scale_fill_manual(values = c("#000271", "#E70007"))+
  scale_x_continuous(name ="Time bins (20 seconds each)" , breaks=seq(1, 6, 1))

ghr
```

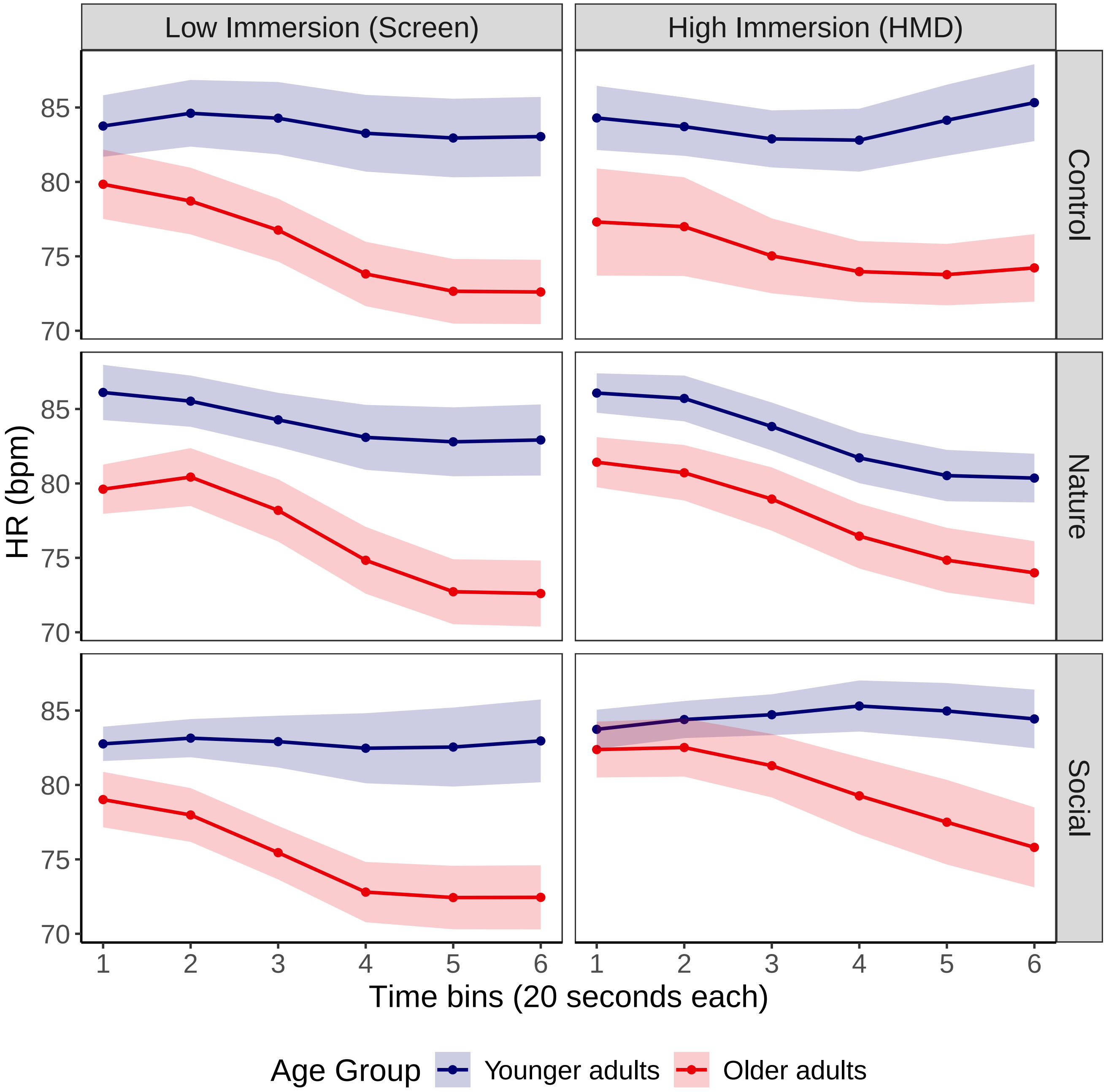

1.6 GCA on HR - Results

Comparisons between a model without any orthogonal polynomial (mHR0), with models containing linear (mHR1), quadratic (mHR2) and cubic (mHR3) time terms.

```
mHR0 <- lmer(value ~ GroupS*ImmersionS*ContentS+
                (1|Sujet),
                      control= lmerControl(optimizer="bobyqa"),
                      na.action = na.exclude,
                      data=HR_long,
                      REML = F)
```

```r
mHR1 <- lmer(value ~ (ot1)*GroupS*ImmersionS*ContentS+
                (ot1||Sujet)+
                (ot1||Sujet:ImmersionS:ContentS),
                      control= lmerControl(optimizer="bobyqa"),
                      na.action = na.exclude,
                      data=HR_long,
                      REML = F)

mHR2 <- lmer(value ~ (ot1+ot2)*GroupS*ImmersionS*ContentS+
                (ot1+ot2||Sujet)+
                (ot1+ot2||Sujet:ImmersionS:ContentS),
                      control= lmerControl(optimizer="bobyqa"),
                      na.action = na.exclude,
                      data=HR_long,
                      REML = F)

mHR3 <- lmer(value ~ (ot1+ot2+ot3)*GroupS*ImmersionS*ContentS+
                (ot1+ot2+ot3||Sujet)+
                (ot1+ot2||Sujet:ImmersionS:ContentS),
                      control= lmerControl(optimizer="bobyqa"),
                      na.action = na.exclude,
                      data=HR_long,
                      REML = F)
```

1.7 Supplementary Table 2. Model comparison

```r
anova(mHR0, mHR1, mHR2, mHR3)
```

```
## Data: HR_long
## Models:
## mHR0: value ~ GroupS * ImmersionS * ContentS + (1 | Sujet)
## mHR1: value ~ (ot1) * GroupS * ImmersionS * ContentS + (ot1 || Sujet) + (ot1 || Sujet:ImmersionS:Conten
## mHR2: value ~ (ot1 + ot2) * GroupS * ImmersionS * ContentS + (ot1 + ot2 || Sujet) + (ot1 + ot2 || Sujet
## mHR3: value ~ (ot1 + ot2 + ot3) * GroupS * ImmersionS * ContentS + (ot1 + ot2 + ot3 || Sujet) + (ot1 +
##      npar   AIC   BIC logLik deviance Chisq Df           Pr(>Chisq)
## mHR0   14 14865 14944  -7418    14837
## mHR1   29 12289 12453  -6115    12231  2606 15 <0.0000000000000002 ***
## mHR2   43 11830 12073  -5872    11744   487 14 <0.0000000000000002 ***
## mHR3   56 11642 11958  -5765    11530   215 13 <0.0000000000000002 ***
## ---
## Signif. codes:  0 '***' 0.001 '**' 0.01 '*' 0.05 '.' 0.1 ' ' 1
```

1.8 Supplementary Table 3. Results of the selected model

Investigation of main effects and interactions within the model containing linear, quadratic and cubic time terms.

```r
anova(mHR3)
```

```
## Type III Analysis of Variance Table with Satterthwaite's method
##                                 Sum Sq Mean Sq NumDF DenDF F value    Pr(>F)
## ot1                               39.1    39.1     1    58   17.00   0.00012 ***
## ot2                                4.8     4.8     1    58    2.10   0.15228
```

```
## ot3                              67.5    67.5     1    58   29.36 0.0000012 ***
## GroupS                           23.5    23.5     1    58   10.22   0.00225 **
## ImmersionS                        5.5     5.5     1   290    2.38   0.12422
## ContentS                          3.0     1.5     2   290    0.64   0.52627
## ot1:GroupS                       15.3    15.3     1    58    6.65   0.01249 *
## ot2:GroupS                        0.0     0.0     1    58    0.00   0.99736
## ot3:GroupS                       11.4    11.4     1    58    4.98   0.02960 *
## ot1:ImmersionS                    1.3     1.3     1   290    0.59   0.44495
## ot2:ImmersionS                    0.5     0.5     1   290    0.22   0.64275
## ot3:ImmersionS                   23.6    23.6     1   986   10.27   0.00139 **
## GroupS:ImmersionS                 2.8     2.8     1   290    1.22   0.26989
## ot1:ContentS                     16.6     8.3     2   290    3.61   0.02832 *
## ot2:ContentS                      9.0     4.5     2   290    1.96   0.14246
## ot3:ContentS                     31.1    15.5     2   986    6.76   0.00121 **
## GroupS:ContentS                   3.7     1.9     2   290    0.81   0.44771
## ImmersionS:ContentS              11.3     5.6     2   290    2.46   0.08736 .
## ot1:GroupS:ImmersionS             1.1     1.1     1   290    0.47   0.49374
## ot2:GroupS:ImmersionS             2.8     2.8     1   290    1.23   0.26885
## ot3:GroupS:ImmersionS             5.2     5.2     1   986    2.28   0.13155
## ot1:GroupS:ContentS               4.5     2.3     2   290    0.98   0.37502
## ot2:GroupS:ContentS               4.4     2.2     2   290    0.96   0.38552
## ot3:GroupS:ContentS               1.6     0.8     2   986    0.34   0.71009
## ot1:ImmersionS:ContentS           5.2     2.6     2   290    1.14   0.32120
## ot2:ImmersionS:ContentS          21.1    10.5     2   290    4.59   0.01092 *
## ot3:ImmersionS:ContentS           1.0     0.5     2   986    0.21   0.80951
## GroupS:ImmersionS:ContentS        3.1     1.5     2   290    0.67   0.51124
## ot1:GroupS:ImmersionS:ContentS    1.2     0.6     2   290    0.26   0.77187
## ot2:GroupS:ImmersionS:ContentS    5.0     2.5     2   290    1.09   0.33903
## ot3:GroupS:ImmersionS:ContentS   23.5    11.7     2   986    5.10   0.00623 **
## ---
## Signif. codes:  0 '***' 0.001 '**' 0.01 '*' 0.05 '.' 0.1 ' ' 1
```

1.9 Supplementary Table 4. Estimates for significant effects and interactions

```r
summary(mHR3)
```

```
## Linear mixed model fit by maximum likelihood . t-tests use Satterthwaite's
##   method [lmerModLmerTest]
## Formula: value ~ (ot1 + ot2 + ot3) * GroupS * ImmersionS * ContentS +
##     (ot1 + ot2 + ot3 || Sujet) + (ot1 + ot2 || Sujet:ImmersionS:ContentS)
##    Data: HR_long
## Control: lmerControl(optimizer = "bobyqa")
##
##      AIC      BIC   logLik deviance df.resid
##    11642    11958    -5765    11530     2032
##
## Scaled residuals:
##    Min     1Q Median     3Q    Max
## -3.689 -0.373 -0.015  0.356  5.292
##
## Random effects:
##  Groups                      Name        Variance Std.Dev.
##  Sujet.ImmersionS.ContentS   ot2          4774.4   69.10
##  Sujet.ImmersionS.ContentS.1 ot1         30686.2  175.17
```

```
##  Sujet.ImmersionS.ContentS.2 (Intercept)    41.3    6.43
##  Sujet                       ot3           409.4   20.23
##  Sujet.1                     ot2            35.8    5.98
##  Sujet.2                     ot1         10771.7  103.79
##  Sujet.3                     (Intercept)    61.1    7.82
##  Residual                                    2.3    1.52
## Number of obs: 2088, groups:  Sujet:ImmersionS:ContentS, 348; Sujet, 58
##
## Fixed effects:
##                                                Estimate Std. Error       df
## (Intercept)                                     80.1630     1.0995  58.0001
## ot1                                            -69.5894    16.8718  57.9994
## ot2                                              5.9955     4.1406  57.9960
## ot3                                             16.8279     3.1055  58.0001
## GroupS                                          -3.5139     1.0995  58.0001
## ImmersionS                                       0.5424     0.3514 290.0000
## ContentS.Nature-intercept                        0.1582     0.4970 290.0000
## ContentS.Social-intercept                        0.3895     0.4970 290.0000
## ot1:GroupS                                     -43.4998    16.8718  57.9994
## ot2:GroupS                                       0.0109     4.1406  57.9960
## ot3:GroupS                                       6.9286     3.1055  58.0001
## ot1:ImmersionS                                   7.3675     9.6565 290.0004
## ot2:ImmersionS                                  -1.9005     4.0630 289.9946
## ot3:ImmersionS                                  -4.9318     1.5390 986.0002
## GroupS:ImmersionS                                0.3890     0.3514 290.0000
## ot1:ContentS.Nature-intercept                  -36.5195    13.6564 290.0004
## ot1:ContentS.Social-intercept                   15.2397    13.6564 290.0004
## ot2:ContentS.Nature-intercept                   -2.5081     5.7460 289.9946
## ot2:ContentS.Social-intercept                   -8.3607     5.7460 289.9946
## ot3:ContentS.Nature-intercept                    7.5713     2.1765 986.0002
## ot3:ContentS.Social-intercept                   -6.0347     2.1765 986.0002
## GroupS:ContentS.Nature-intercept                 0.2576     0.4970 290.0000
## GroupS:ContentS.Social-intercept                 0.3700     0.4970 290.0000
## ImmersionS:ContentS.Nature-intercept            -0.4807     0.4970 290.0000
## ImmersionS:ContentS.Social-intercept             1.0991     0.4970 290.0000
## ot1:GroupS:ImmersionS                            6.6151     9.6565 290.0004
## ot2:GroupS:ImmersionS                           -4.4961     4.0630 289.9946
## ot3:GroupS:ImmersionS                           -2.3262     1.5390 986.0002
## ot1:GroupS:ContentS.Nature-intercept            17.1783    13.6564 290.0004
## ot1:GroupS:ContentS.Social-intercept           -15.9362    13.6564 290.0004
## ot2:GroupS:ContentS.Nature-intercept            -7.8558     5.7460 289.9946
## ot2:GroupS:ContentS.Social-intercept             4.9652     5.7460 289.9946
## ot3:GroupS:ContentS.Nature-intercept             0.7759     2.1765 986.0002
## ot3:GroupS:ContentS.Social-intercept             1.0199     2.1765 986.0002
## ot1:ImmersionS:ContentS.Nature-intercept       -16.6466    13.6564 290.0004
## ot1:ImmersionS:ContentS.Social-intercept        -2.2182    13.6564 290.0004
## ot2:ImmersionS:ContentS.Nature-intercept         2.1466     5.7460 289.9946
## ot2:ImmersionS:ContentS.Social-intercept       -16.0331     5.7460 289.9946
## ot3:ImmersionS:ContentS.Nature-intercept         1.4142     2.1765 986.0002
## ot3:ImmersionS:ContentS.Social-intercept        -0.6612     2.1765 986.0002
## GroupS:ImmersionS:ContentS.Nature-intercept      0.2156     0.4970 290.0000
## GroupS:ImmersionS:ContentS.Social-intercept      0.3551     0.4970 290.0000
## ot1:GroupS:ImmersionS:ContentS.Nature-intercept   7.4039    13.6564 290.0004
## ot1:GroupS:ImmersionS:ContentS.Social-intercept  -9.3043    13.6564 290.0004
## ot2:GroupS:ImmersionS:ContentS.Nature-intercept   8.3980     5.7460 289.9946
## ot2:GroupS:ImmersionS:ContentS.Social-intercept  -3.2642     5.7460 289.9946
```

```
## ot3:GroupS:ImmersionS:ContentS.Nature-intercept  -6.9528     2.1765 986.0002
## ot3:GroupS:ImmersionS:ContentS.Social-intercept   3.5860     2.1765 986.0002
##                                                  t value            Pr(>|t|)
## (Intercept)                                        72.91 < 0.0000000000000002
## ot1                                                -4.12             0.00012
## ot2                                                 1.45             0.15301
## ot3                                                 5.42           0.0000012
## GroupS                                             -3.20             0.00226
## ImmersionS                                          1.54             0.12379
## ContentS.Nature-intercept                           0.32             0.75047
## ContentS.Social-intercept                           0.78             0.43384
## ot1:GroupS                                         -2.58             0.01249
## ot2:GroupS                                          0.00             0.99792
## ot3:GroupS                                          2.23             0.02956
## ot1:ImmersionS                                      0.76             0.44611
## ot2:ImmersionS                                     -0.47             0.64030
## ot3:ImmersionS                                     -3.20             0.00140
## GroupS:ImmersionS                                   1.11             0.26919
## ot1:ContentS.Nature-intercept                      -2.67             0.00792
## ot1:ContentS.Social-intercept                       1.12             0.26537
## ot2:ContentS.Nature-intercept                      -0.44             0.66281
## ot2:ContentS.Social-intercept                      -1.46             0.14674
## ot3:ContentS.Nature-intercept                       3.48             0.00053
## ot3:ContentS.Social-intercept                      -2.77             0.00567
## GroupS:ContentS.Nature-intercept                    0.52             0.60462
## GroupS:ContentS.Social-intercept                    0.74             0.45721
## ImmersionS:ContentS.Nature-intercept               -0.97             0.33420
## ImmersionS:ContentS.Social-intercept                2.21             0.02778
## ot1:GroupS:ImmersionS                               0.69             0.49387
## ot2:GroupS:ImmersionS                              -1.11             0.26939
## ot3:GroupS:ImmersionS                              -1.51             0.13099
## ot1:GroupS:ContentS.Nature-intercept                1.26             0.20944
## ot1:GroupS:ContentS.Social-intercept               -1.17             0.24419
## ot2:GroupS:ContentS.Nature-intercept               -1.37             0.17263
## ot2:GroupS:ContentS.Social-intercept                0.86             0.38824
## ot3:GroupS:ContentS.Nature-intercept                0.36             0.72157
## ot3:GroupS:ContentS.Social-intercept                0.47             0.63945
## ot1:ImmersionS:ContentS.Nature-intercept           -1.22             0.22385
## ot1:ImmersionS:ContentS.Social-intercept           -0.16             0.87108
## ot2:ImmersionS:ContentS.Nature-intercept            0.37             0.70899
## ot2:ImmersionS:ContentS.Social-intercept           -2.79             0.00561
## ot3:ImmersionS:ContentS.Nature-intercept            0.65             0.51601
## ot3:ImmersionS:ContentS.Social-intercept           -0.30             0.76135
## GroupS:ImmersionS:ContentS.Nature-intercept         0.43             0.66469
## GroupS:ImmersionS:ContentS.Social-intercept         0.71             0.47553
## ot1:GroupS:ImmersionS:ContentS.Nature-intercept     0.54             0.58813
## ot1:GroupS:ImmersionS:ContentS.Social-intercept    -0.68             0.49621
## ot2:GroupS:ImmersionS:ContentS.Nature-intercept     1.46             0.14495
## ot2:GroupS:ImmersionS:ContentS.Social-intercept    -0.57             0.57042
## ot3:GroupS:ImmersionS:ContentS.Nature-intercept    -3.19             0.00145
## ot3:GroupS:ImmersionS:ContentS.Social-intercept     1.65             0.09976
##
## (Intercept)                                      ***
## ot1                                              ***
## ot2
## ot3                                              ***
```

```
## GroupS                                          **
## ImmersionS
## ContentS.Nature-intercept
## ContentS.Social-intercept
## ot1:GroupS                                      *
## ot2:GroupS
## ot3:GroupS                                      *
## ot1:ImmersionS
## ot2:ImmersionS
## ot3:ImmersionS                                  **
## GroupS:ImmersionS
## ot1:ContentS.Nature-intercept                   **
## ot1:ContentS.Social-intercept
## ot2:ContentS.Nature-intercept
## ot2:ContentS.Social-intercept
## ot3:ContentS.Nature-intercept                   ***
## ot3:ContentS.Social-intercept                   **
## GroupS:ContentS.Nature-intercept
## GroupS:ContentS.Social-intercept
## ImmersionS:ContentS.Nature-intercept
## ImmersionS:ContentS.Social-intercept            *
## ot1:GroupS:ImmersionS
## ot2:GroupS:ImmersionS
## ot3:GroupS:ImmersionS
## ot1:GroupS:ContentS.Nature-intercept
## ot1:GroupS:ContentS.Social-intercept
## ot2:GroupS:ContentS.Nature-intercept
## ot2:GroupS:ContentS.Social-intercept
## ot3:GroupS:ContentS.Nature-intercept
## ot3:GroupS:ContentS.Social-intercept
## ot1:ImmersionS:ContentS.Nature-intercept
## ot1:ImmersionS:ContentS.Social-intercept
## ot2:ImmersionS:ContentS.Nature-intercept
## ot2:ImmersionS:ContentS.Social-intercept        **
## ot3:ImmersionS:ContentS.Nature-intercept
## ot3:ImmersionS:ContentS.Social-intercept
## GroupS:ImmersionS:ContentS.Nature-intercept
## GroupS:ImmersionS:ContentS.Social-intercept
## ot1:GroupS:ImmersionS:ContentS.Nature-intercept
## ot1:GroupS:ImmersionS:ContentS.Social-intercept
## ot2:GroupS:ImmersionS:ContentS.Nature-intercept
## ot2:GroupS:ImmersionS:ContentS.Social-intercept
## ot3:GroupS:ImmersionS:ContentS.Nature-intercept **
## ot3:GroupS:ImmersionS:ContentS.Social-intercept .
## ---
## Signif. codes:  0 '***' 0.001 '**' 0.01 '*' 0.05 '.' 0.1 ' ' 1

##
## Correlation matrix not shown by default, as p = 48 > 12.
## Use print(x, correlation=TRUE)  or
##     vcov(x)        if you need it
```

1.9.1 GCA on HR for Screen

```r
mHRSC <- lmer(value ~ (ot1+ot2+ot3)*GroupS*ContentS+
                (ot1+ot2+ot3||Sujet)+
                (ot1+ot2||Sujet:ContentS),
                      control= lmerControl(optimizer="bobyqa"),
                      na.action = na.exclude,
                      data=subset(HR_long, Immersion=="Low Immersion (Screen)"),
                      REML = F)
```

```r
anova(mHRSC)
```

1.9.1.1 Supplementary Table 5. Investigation of main effects and interactions

```
## Type III Analysis of Variance Table with Satterthwaite's method
##                      Sum Sq Mean Sq NumDF DenDF F value     Pr(>F)
## ot1                    26.9    26.9     1    58   13.88   0.00044 ***
## ot2                     3.0     3.0     1    58    1.52   0.22216
## ot3                    50.8    50.8     1    58   26.23 0.0000036 ***
## GroupS                 16.9    16.9     1    58    8.74   0.00449 **
## ContentS                4.4     2.2     2   116    1.14   0.32483
## ot1:GroupS             11.4    11.4     1    58    5.89   0.01839 *
## ot2:GroupS              1.0     1.0     1    58    0.50   0.48307
## ot3:GroupS              9.2     9.2     1    58    4.74   0.03354 *
## ot1:ContentS            3.7     1.8     2   116    0.95   0.39075
## ot2:ContentS            1.8     0.9     2   116    0.48   0.62267
## ot3:ContentS           10.9     5.4     2   464    2.81   0.06106 .
## GroupS:ContentS         0.0     0.0     2   116    0.01   0.99356
## ot1:GroupS:ContentS     0.8     0.4     2   116    0.20   0.81853
## ot2:GroupS:ContentS     8.1     4.1     2   116    2.10   0.12671
## ot3:GroupS:ContentS    15.0     7.5     2   464    3.88   0.02132 *
## ---
## Signif. codes:  0 '***' 0.001 '**' 0.01 '*' 0.05 '.' 0.1 ' ' 1
```

```r
summary(mHRSC)
```

1.9.1.2 Supplementary Table 6. Estimates for significant effects and interactions

```
## Linear mixed model fit by maximum likelihood . t-tests use Satterthwaite's
##   method [lmerModLmerTest]
## Formula: value ~ (ot1 + ot2 + ot3) * GroupS * ContentS + (ot1 + ot2 +
##     ot3 || Sujet) + (ot1 + ot2 || Sujet:ContentS)
##    Data: subset(HR_long, Immersion == "Low Immersion (Screen)")
## Control: lmerControl(optimizer = "bobyqa")
##
##      AIC      BIC   logLik deviance df.resid
##     5737     5896    -2837     5673     1012
##
## Scaled residuals:
##    Min     1Q Median     3Q    Max
## -3.413 -0.351  0.001  0.337  3.249
```

```
##
## Random effects:
##  Groups           Name        Variance Std.Dev.
##  Sujet.ContentS   ot2          4628.10  68.03
##  Sujet.ContentS.1 ot1         20281.84 142.41
##  Sujet.ContentS.2 (Intercept)    22.40   4.73
##  Sujet            ot3           790.93  28.12
##  Sujet.1          ot2           534.73  23.12
##  Sujet.2          ot1         17029.55 130.50
##  Sujet.3          (Intercept)    90.46   9.51
##  Residual                         1.94   1.39
## Number of obs: 1044, groups:  Sujet:ContentS, 174; Sujet, 58
##
## Fixed effects:
##                                     Estimate Std. Error       df t value
## (Intercept)                          79.6206     1.3198  58.0000   60.33
## ot1                                 -76.9569    20.6576  57.9999   -3.73
## ot2                                   7.8961     6.3960  58.0000    1.23
## ot3                                  21.7597     4.2483  58.0000    5.12
## GroupS                               -3.9029     1.3198  58.0000   -2.96
## ContentS.Nature-intercept             0.6389     0.5188 116.0000    1.23
## ContentS.Social-intercept            -0.7096     0.5188 116.0000   -1.37
## ot1:GroupS                          -50.1149    20.6576  57.9999   -2.43
## ot2:GroupS                            4.5069     6.3960  58.0000    0.70
## ot3:GroupS                            9.2548     4.2483  58.0000    2.18
## ot1:ContentS.Nature-intercept       -19.8729    15.7561 116.0005   -1.26
## ot1:ContentS.Social-intercept        17.4579    15.7561 116.0005    1.11
## ot2:ContentS.Nature-intercept        -4.6546     7.9255 115.9998   -0.59
## ot2:ContentS.Social-intercept         7.6724     7.9255 115.9998    0.97
## ot3:ContentS.Nature-intercept         6.1571     2.8262 463.9998    2.18
## ot3:ContentS.Social-intercept        -5.3735     2.8262 463.9998   -1.90
## GroupS:ContentS.Nature-intercept      0.0420     0.5188 116.0000    0.08
## GroupS:ContentS.Social-intercept      0.0149     0.5188 116.0000    0.03
## ot1:GroupS:ContentS.Nature-intercept   9.7744    15.7561 116.0005    0.62
## ot1:GroupS:ContentS.Social-intercept  -6.6318    15.7561 116.0005   -0.42
## ot2:GroupS:ContentS.Nature-intercept -16.2538     7.9255 115.9998   -2.05
## ot2:GroupS:ContentS.Social-intercept   8.2294     7.9255 115.9998    1.04
## ot3:GroupS:ContentS.Nature-intercept   7.7287     2.8262 463.9998    2.73
## ot3:GroupS:ContentS.Social-intercept  -2.5660     2.8262 463.9998   -0.91
##                                                 Pr(>|t|)
## (Intercept)                          < 0.0000000000000002 ***
## ot1                                              0.00044 ***
## ot2                                              0.22198
## ot3                                            0.0000036 ***
## GroupS                                           0.00449 **
## ContentS.Nature-intercept                        0.22060
## ContentS.Social-intercept                        0.17401
## ot1:GroupS                                       0.01840 *
## ot2:GroupS                                       0.48385
## ot3:GroupS                                       0.03345 *
## ot1:ContentS.Nature-intercept                    0.20974
## ot1:ContentS.Social-intercept                    0.27015
## ot2:ContentS.Nature-intercept                    0.55814
## ot2:ContentS.Social-intercept                    0.33502
## ot3:ContentS.Nature-intercept                    0.02986 *
## ot3:ContentS.Social-intercept                    0.05788 .
```

```
## GroupS:ContentS.Nature-intercept                  0.93567
## GroupS:ContentS.Social-intercept                  0.97712
## ot1:GroupS:ContentS.Nature-intercept              0.53624
## ot1:GroupS:ContentS.Social-intercept              0.67460
## ot2:GroupS:ContentS.Nature-intercept              0.04254 *
## ot2:GroupS:ContentS.Social-intercept              0.30127
## ot3:GroupS:ContentS.Nature-intercept              0.00648 **
## ot3:GroupS:ContentS.Social-intercept              0.36437
## ---
## Signif. codes:  0 '***' 0.001 '**' 0.01 '*' 0.05 '.' 0.1 ' ' 1
```

```
##
## Correlation matrix not shown by default, as p = 24 > 12.
## Use print(x, correlation=TRUE)  or
##     vcov(x)        if you need it
```

1.9.2 GCA on HR for VR

```
mHRVR <- lmer(value ~ (ot1+ot2+ot3)*GroupS*ContentS+
                (ot1+ot2+ot3||Sujet)+
                (ot1+ot2||Sujet:ContentS),
                      control= lmerControl(optimizer="bobyqa"),
                      na.action = na.exclude,
                      data=subset(HR_long, Immersion=="High Immersion (HMD)"),
                      REML = F)
```

```
## boundary (singular) fit: see help('isSingular')
```

```
anova(mHRVR)
```

1.9.2.1 Supplementary Table 7. Investigation of main effects and interactions

```
## Type III Analysis of Variance Table with Satterthwaite's method
##                     Sum Sq Mean Sq NumDF DenDF F value Pr(>F)
## ot1                  19.97   19.97     1    58    9.96 0.0025 **
## ot2                   1.07    1.07     1   174    0.53 0.4666
## ot3                  16.92   16.92     1    58    8.44 0.0052 **
## GroupS               19.00   19.00     1    58    9.48 0.0032 **
## ContentS              8.06    4.03     2   116    2.01 0.1386
## ot1:GroupS            7.03    7.03     1    58    3.50 0.0662 .
## ot2:GroupS            1.27    1.27     1   174    0.63 0.4279
## ot3:GroupS            2.54    2.54     1    58    1.26 0.2654
## ot1:ContentS         14.04    7.02     2   116    3.50 0.0334 *
## ot2:ContentS         25.32   12.66     2   174    6.31 0.0023 **
## ot3:ContentS         21.15   10.58     2   464    5.28 0.0054 **
## GroupS:ContentS       4.79    2.39     2   116    1.19 0.3068
## ot1:GroupS:ContentS   3.78    1.89     2   116    0.94 0.3921
## ot2:GroupS:ContentS   0.17    0.09     2   174    0.04 0.9580
## ot3:GroupS:ContentS  10.00    5.00     2   464    2.49 0.0837 .
## ---
## Signif. codes:  0 '***' 0.001 '**' 0.01 '*' 0.05 '.' 0.1 ' ' 1
```

```r
summary(mHRVR)
```

1.9.2.2 Supplementary Table 8. Estimates for significant effects and interactions

```
## Linear mixed model fit by maximum likelihood . t-tests use Satterthwaite's
##   method [lmerModLmerTest]
## Formula: value ~ (ot1 + ot2 + ot3) * GroupS * ContentS + (ot1 + ot2 +
##     ot3 || Sujet) + (ot1 + ot2 || Sujet:ContentS)
##    Data: subset(HR_long, Immersion == "High Immersion (HMD)")
## Control: lmerControl(optimizer = "bobyqa")
##
##      AIC      BIC   logLik deviance df.resid
##     5861     6020    -2899     5797     1012
##
## Scaled residuals:
##    Min     1Q Median     3Q    Max
## -3.281 -0.361 -0.008  0.350  4.588
##
## Random effects:
##  Groups           Name        Variance          Std.Dev.
##  Sujet.ContentS   ot2          4685.06633211687  68.447544
##  Sujet.ContentS.1 ot1         36327.46722787863 190.597658
##  Sujet.ContentS.2 (Intercept)    51.18250116244   7.154195
##  Sujet            ot3           710.58951313305  26.656885
##  Sujet.1          ot2             0.00000000017   0.000013
##  Sujet.2          ot1          9504.13151852821  97.489135
##  Sujet.3          (Intercept)    40.86511588195   6.392583
##  Residual                         2.00499680022   1.415979
## Number of obs: 1044, groups:  Sujet:ContentS, 174; Sujet, 58
##
## Fixed effects:
##                                      Estimate Std. Error      df t value
## (Intercept)                            80.705      1.016  58.000   79.47
## ot1                                   -62.222     19.703  58.000   -3.16
## ot2                                     4.095      5.647 174.000    0.73
## ot3                                    11.896      4.094  58.000    2.91
## GroupS                                 -3.125      1.016  58.000   -3.08
## ContentS.Nature-intercept              -0.323      0.781 116.000   -0.41
## ContentS.Social-intercept               1.489      0.781 116.000    1.91
## ot1:GroupS                            -36.885     19.703  58.000   -1.87
## ot2:GroupS                             -4.485      5.647 174.000   -0.79
## ot3:GroupS                              4.602      4.094  58.000    1.12
## ot1:ContentS.Nature-intercept         -53.166     20.943 116.001   -2.54
## ot1:ContentS.Social-intercept          13.022     20.943 116.001    0.62
## ot2:ContentS.Nature-intercept          -0.362      7.985 174.000   -0.05
## ot2:ContentS.Social-intercept         -24.394      7.985 174.000   -3.05
## ot3:ContentS.Nature-intercept           8.985      2.875 464.000    3.13
## ot3:ContentS.Social-intercept          -6.696      2.875 464.000   -2.33
## GroupS:ContentS.Nature-intercept        0.473      0.781 116.000    0.61
## GroupS:ContentS.Social-intercept        0.725      0.781 116.000    0.93
## ot1:GroupS:ContentS.Nature-intercept   24.582     20.943 116.001    1.17
## ot1:GroupS:ContentS.Social-intercept  -25.241     20.943 116.001   -1.21
## ot2:GroupS:ContentS.Nature-intercept    0.542      7.985 174.000    0.07
## ot2:GroupS:ContentS.Social-intercept    1.701      7.985 174.000    0.21
```

```
## ot3:GroupS:ContentS.Nature-intercept   -6.177      2.875 464.000   -2.15
## ot3:GroupS:ContentS.Social-intercept    4.606      2.875 464.000    1.60
##                                                     Pr(>|t|)
## (Intercept)                          <0.0000000000000002 ***
## ot1                                               0.0025 **
## ot2                                               0.4693
## ot3                                               0.0052 **
## GroupS                                            0.0032 **
## ContentS.Nature-intercept                         0.6805
## ContentS.Social-intercept                         0.0592 .
## ot1:GroupS                                        0.0662 .
## ot2:GroupS                                        0.4281
## ot3:GroupS                                        0.2656
## ot1:ContentS.Nature-intercept                     0.0125 *
## ot1:ContentS.Social-intercept                     0.5353
## ot2:ContentS.Nature-intercept                     0.9639
## ot2:ContentS.Social-intercept                     0.0026 **
## ot3:ContentS.Nature-intercept                     0.0019 **
## ot3:ContentS.Social-intercept                     0.0203 *
## GroupS:ContentS.Nature-intercept                  0.5458
## GroupS:ContentS.Social-intercept                  0.3553
## ot1:GroupS:ContentS.Nature-intercept              0.2429
## ot1:GroupS:ContentS.Social-intercept              0.2306
## ot2:GroupS:ContentS.Nature-intercept              0.9459
## ot2:GroupS:ContentS.Social-intercept              0.8316
## ot3:GroupS:ContentS.Nature-intercept              0.0322 *
## ot3:GroupS:ContentS.Social-intercept              0.1098
## ---
## Signif. codes:  0 '***' 0.001 '**' 0.01 '*' 0.05 '.' 0.1 ' ' 1

##
## Correlation matrix not shown by default, as p = 24 > 12.
## Use print(x, correlation=TRUE)  or
##     vcov(x)        if you need it

## optimizer (bobyqa) convergence code: 0 (OK)
## boundary (singular) fit: see help('isSingular')
```

2 GCA on Skin Conductance Level (SCL)

2.1 Dataframe manipulation

```
SCL$`Age Group` <- as.factor(SCL$Groupe)
SCL = subset(SCL, select = -c(Groupe) )
str(SCL)
```

```
## 'data.frame':    58 obs. of  38 variables:
##  $ Sujet    : chr  "AB_090221" "AGM_090321" "AG_090321" "AM_230221" ...
##  $ EC_Tuto_1: num  0.31 0.249 0.143 0.284 0.476 ...
##  $ EC_Tuto_2: num  0.301 0.249 0.208 0.274 0.456 ...
##  $ EC_Tuto_3: num  0.261 0.25 0.291 0.202 0.433 ...
##  $ EC_Tuto_4: num  0.22 0.252 0.436 0.147 0.413 ...
##  $ EC_Tuto_5: num  0.1894 0.2503 0.7452 0.0997 0.4053 ...
```

```
##  $ EC_Tuto_6: num  0.1735 0.2465 0.9205 0.0485 0.3911 ...
##  $ EC_NS_1  : num  0.118 0.287 0.12 0.382 0.599 ...
##  $ EC_NS_2  : num  0.102 0.283 0.144 0.402 0.573 ...
##  $ EC_NS_3  : num  0.0822 0.2749 0.1561 0.3667 0.524 ...
##  $ EC_NS_4  : num  0.0667 0.2718 0.1638 0.3232 0.4751 ...
##  $ EC_NS_5  : num  0.0533 0.2709 0.1699 0.2699 0.4887 ...
##  $ EC_NS_6  : num  0.0463 0.2677 0.1735 0.2234 0.4164 ...
##  $ EC_S_1   : num  0.0634 0.3397 0.1406 0.2823 0.6002 ...
##  $ EC_S_2   : num  0.0739 0.3422 0.1558 0.2471 0.5779 ...
##  $ EC_S_3   : num  0.0732 0.3455 0.1635 0.2432 0.5633 ...
##  $ EC_S_4   : num  0.0901 0.335 0.1706 0.1928 0.5402 ...
##  $ EC_S_5   : num  0.085 0.326 0.179 0.183 0.529 ...
##  $ EC_S_6   : num  0.0714 0.3146 0.1823 0.2574 0.4751 ...
##  $ VR_Tuto_1: num  0.617 0.921 0.364 0.179 0.978 ...
##  $ VR_Tuto_2: num  0.638 0.747 0.372 0.199 0.976 ...
##  $ VR_Tuto_3: num  0.602 0.594 0.447 0.153 0.899 ...
##  $ VR_Tuto_4: num  0.606 0.465 0.423 0.0959 0.8641 ...
##  $ VR_Tuto_5: num  0.6378 0.3244 0.4913 0.0488 0.8032 ...
##  $ VR_Tuto_6: num  0.5913 0.2059 0.4728 0.0184 0.7595 ...
##  $ VR_NS_1  : num  0.305 0.175 0.182 0.819 0.531 ...
##  $ VR_NS_2  : num  0.286 0.196 0.191 0.738 0.573 ...
##  $ VR_NS_3  : num  0.285 0.203 0.203 0.656 0.555 ...
##  $ VR_NS_4  : num  0.283 0.194 0.209 0.684 0.53 ...
##  $ VR_NS_5  : num  0.291 0.185 0.24 0.74 0.492 ...
##  $ VR_NS_6  : num  0.28 0.172 0.303 0.726 0.428 ...
##  $ VR_S_1   : num  0.325 0.168 0.221 0.776 0.548 ...
##  $ VR_S_2   : num  0.333 0.181 0.206 0.754 0.503 ...
##  $ VR_S_3   : num  0.287 0.19 0.203 0.692 0.444 ...
##  $ VR_S_4   : num  0.341 0.194 0.204 0.632 0.557 ...
##  $ VR_S_5   : num  0.465 0.178 0.206 0.659 0.414 ...
##  $ VR_S_6   : num  0.483 0.157 0.216 0.659 0.379 ...
##  $ Age Group: Factor w/ 2 levels "JA","PA": 1 1 1 1 1 1 1 1 1 1 ...
```

2.1.1 Pivot dataframe from wide to long format

```
SCL_long <- SCL %>%
  pivot_longer(names_to = c("Immersion","Content","bin"),
               names_sep = "_",
               -c(Sujet, `Age Group`))
```

2.1.2 Define factors and their levels

```
SCL_long$`Age Group` <- as.factor(SCL_long$`Age Group`)
SCL_long$`Age Group` <- revalue(SCL_long$`Age Group`, c("JA"= "Younger adults", "PA" = "Older adults"))
SCL_long$bin <- as.numeric(SCL_long$bin)
SCL_long$Immersion <- as.factor(SCL_long$Immersion)
SCL_long$Immersion <- revalue(SCL_long$Immersion, c("EC"="Low Immersion (Screen)", "VR"= "High Immersion (
SCL_long$Content <- revalue(SCL_long$Content, c("Tuto"="Control", "NS"= "Nature", "S" = "Social"))
SCL_long$Content <- factor(SCL_long$Content, levels = c("Control", "Nature", "Social"))
SCL_long$Sujet <- as.factor(SCL_long$Sujet)
```

2.2 Creation of the polynomials

```
polynomials <- poly(SCL_long$bin, degree = 3)
SCL_long$ot1 <- polynomials[,1]
SCL_long$ot2 <- polynomials[,2]
SCL_long$ot3 <- polynomials[,3]
```

2.3 Creation of the contrasts (Sum coding)

```
SCL_long$ContentS <- contr_code_sum(SCL_long$Content, omit = 1)
SCL_long$GroupS <- ifelse(SCL_long$`Age Group` == "Older adults", 1, -1)
SCL_long$ImmersionS <- ifelse(SCL_long$Immersion == "High Immersion (HMD)", 1, -1)
str(SCL_long)
```

```
## tibble [2,088 x 12] (S3: tbl_df/tbl/data.frame)
##  $ Sujet     : Factor w/ 58 levels "AA_230321","AB_090221",..: 2 2 2 2 2 2 2 2 2 2 ...
##  $ Age Group : Factor w/ 2 levels "Younger adults",..: 1 1 1 1 1 1 1 1 1 1 ...
##  $ Immersion : Factor w/ 2 levels "Low Immersion (Screen)",..: 1 1 1 1 1 1 1 1 1 1 ...
##  $ Content   : Factor w/ 3 levels "Control","Nature",..: 1 1 1 1 1 1 2 2 2 2 ...
##  $ bin       : num [1:2088] 1 2 3 4 5 6 1 2 3 4 ...
##  $ value     : num [1:2088] 0.31 0.301 0.261 0.22 0.189 ...
##  $ ot1       : num [1:2088] -0.03204 -0.01922 -0.00641 0.00641 0.01922 ...
##  $ ot2       : num [1:2088] 0.02924 -0.00585 -0.0234 -0.0234 -0.00585 ...
##  $ ot3       : num [1:2088] -0.02 0.028 0.016 -0.016 -0.028 ...
##  $ ContentS  : Factor w/ 3 levels "Control","Nature",..: 1 1 1 1 1 1 2 2 2 2 ...
##   ..- attr(*, "contrasts")= num [1:3, 1:2] -1 1 0 -1 0 1
##   .. ..- attr(*, "dimnames")=List of 2
##   .. .. ..$ : chr [1:3] "Control" "Nature" "Social"
##   .. .. ..$ : chr [1:2] ".Nature-intercept" ".Social-intercept"
##  $ GroupS    : num [1:2088] -1 -1 -1 -1 -1 -1 -1 -1 -1 -1 ...
##  $ ImmersionS: num [1:2088] -1 -1 -1 -1 -1 -1 -1 -1 -1 -1 ...
```

2.4 Supplementary Table 9. Descriptive statistics for SCL data

```
mseSCL <- ddply(SCL_long, c("`Age Group`", "Immersion", "Content","bin"), summarise,
               N    = length(unique(Sujet)),
               mean = mean(value, na.rm=TRUE),
               sd   = sd(value, na.rm = TRUE),
               se   = sd / sqrt(N))


mseSCL
```

```
##         Age Group              Immersion Content bin  N  mean    sd     se
## 1  Younger adults Low Immersion (Screen) Control   1 35 0.363 0.266 0.0450
## 2  Younger adults Low Immersion (Screen) Control   2 35 0.397 0.277 0.0468
## 3  Younger adults Low Immersion (Screen) Control   3 35 0.405 0.279 0.0472
## 4  Younger adults Low Immersion (Screen) Control   4 35 0.407 0.286 0.0484
## 5  Younger adults Low Immersion (Screen) Control   5 35 0.421 0.303 0.0513
## 6  Younger adults Low Immersion (Screen) Control   6 35 0.428 0.316 0.0534
```

```
## 7  Younger adults Low Immersion (Screen)  Nature   1 35 0.391 0.256 0.0433
## 8  Younger adults Low Immersion (Screen)  Nature   2 35 0.401 0.265 0.0449
## 9  Younger adults Low Immersion (Screen)  Nature   3 35 0.390 0.268 0.0454
## 10 Younger adults Low Immersion (Screen)  Nature   4 35 0.387 0.272 0.0459
## 11 Younger adults Low Immersion (Screen)  Nature   5 35 0.378 0.273 0.0461
## 12 Younger adults Low Immersion (Screen)  Nature   6 35 0.365 0.270 0.0456
## 13 Younger adults Low Immersion (Screen)  Social   1 35 0.366 0.240 0.0406
## 14 Younger adults Low Immersion (Screen)  Social   2 35 0.377 0.254 0.0430
## 15 Younger adults Low Immersion (Screen)  Social   3 35 0.374 0.251 0.0424
## 16 Younger adults Low Immersion (Screen)  Social   4 35 0.370 0.251 0.0424
## 17 Younger adults Low Immersion (Screen)  Social   5 35 0.374 0.255 0.0430
## 18 Younger adults Low Immersion (Screen)  Social   6 35 0.378 0.259 0.0438
## 19 Younger adults   High Immersion (HMD) Control   1 35 0.494 0.296 0.0501
## 20 Younger adults   High Immersion (HMD) Control   2 35 0.524 0.260 0.0439
## 21 Younger adults   High Immersion (HMD) Control   3 35 0.507 0.268 0.0454
## 22 Younger adults   High Immersion (HMD) Control   4 35 0.507 0.298 0.0503
## 23 Younger adults   High Immersion (HMD) Control   5 35 0.510 0.312 0.0527
## 24 Younger adults   High Immersion (HMD) Control   6 35 0.488 0.309 0.0523
## 25 Younger adults   High Immersion (HMD)  Nature   1 35 0.382 0.208 0.0352
## 26 Younger adults   High Immersion (HMD)  Nature   2 35 0.400 0.224 0.0378
## 27 Younger adults   High Immersion (HMD)  Nature   3 35 0.409 0.226 0.0382
## 28 Younger adults   High Immersion (HMD)  Nature   4 35 0.396 0.225 0.0380
## 29 Younger adults   High Immersion (HMD)  Nature   5 35 0.392 0.226 0.0382
## 30 Younger adults   High Immersion (HMD)  Nature   6 35 0.387 0.223 0.0377
## 31 Younger adults   High Immersion (HMD)  Social   1 35 0.340 0.216 0.0365
## 32 Younger adults   High Immersion (HMD)  Social   2 35 0.348 0.197 0.0333
## 33 Younger adults   High Immersion (HMD)  Social   3 35 0.371 0.204 0.0344
## 34 Younger adults   High Immersion (HMD)  Social   4 35 0.398 0.214 0.0361
## 35 Younger adults   High Immersion (HMD)  Social   5 35 0.425 0.222 0.0376
## 36 Younger adults   High Immersion (HMD)  Social   6 35 0.444 0.223 0.0376
## 37   Older adults Low Immersion (Screen) Control   1 23 0.343 0.271 0.0565
## 38   Older adults Low Immersion (Screen) Control   2 23 0.418 0.317 0.0661
## 39   Older adults Low Immersion (Screen) Control   3 23 0.426 0.332 0.0692
## 40   Older adults Low Immersion (Screen) Control   4 23 0.421 0.333 0.0694
## 41   Older adults Low Immersion (Screen) Control   5 23 0.407 0.327 0.0682
## 42   Older adults Low Immersion (Screen) Control   6 23 0.392 0.324 0.0675
## 43   Older adults Low Immersion (Screen)  Nature   1 23 0.330 0.218 0.0454
## 44   Older adults Low Immersion (Screen)  Nature   2 23 0.335 0.187 0.0390
## 45   Older adults Low Immersion (Screen)  Nature   3 23 0.331 0.178 0.0372
## 46   Older adults Low Immersion (Screen)  Nature   4 23 0.309 0.168 0.0350
## 47   Older adults Low Immersion (Screen)  Nature   5 23 0.305 0.179 0.0373
## 48   Older adults Low Immersion (Screen)  Nature   6 23 0.272 0.164 0.0342
## 49   Older adults Low Immersion (Screen)  Social   1 23 0.290 0.187 0.0390
## 50   Older adults Low Immersion (Screen)  Social   2 23 0.293 0.182 0.0380
## 51   Older adults Low Immersion (Screen)  Social   3 23 0.289 0.172 0.0359
## 52   Older adults Low Immersion (Screen)  Social   4 23 0.274 0.156 0.0325
## 53   Older adults Low Immersion (Screen)  Social   5 23 0.258 0.158 0.0330
## 54   Older adults Low Immersion (Screen)  Social   6 23 0.253 0.165 0.0343
## 55   Older adults   High Immersion (HMD) Control   1 23 0.497 0.335 0.0698
## 56   Older adults   High Immersion (HMD) Control   2 23 0.509 0.342 0.0712
## 57   Older adults   High Immersion (HMD) Control   3 23 0.498 0.332 0.0692
## 58   Older adults   High Immersion (HMD) Control   4 23 0.476 0.321 0.0670
## 59   Older adults   High Immersion (HMD) Control   5 23 0.465 0.320 0.0667
## 60   Older adults   High Immersion (HMD) Control   6 23 0.457 0.318 0.0662
## 61   Older adults   High Immersion (HMD)  Nature   1 23 0.446 0.237 0.0495
## 62   Older adults   High Immersion (HMD)  Nature   2 23 0.479 0.242 0.0504
```

```
## 63   Older adults   High Immersion (HMD)  Nature   3 23 0.488 0.235 0.0489
## 64   Older adults   High Immersion (HMD)  Nature   4 23 0.481 0.228 0.0476
## 65   Older adults   High Immersion (HMD)  Nature   5 23 0.468 0.225 0.0469
## 66   Older adults   High Immersion (HMD)  Nature   6 23 0.448 0.228 0.0474
## 67   Older adults   High Immersion (HMD)  Social   1 23 0.482 0.209 0.0437
## 68   Older adults   High Immersion (HMD)  Social   2 23 0.520 0.223 0.0465
## 69   Older adults   High Immersion (HMD)  Social   3 23 0.514 0.241 0.0502
## 70   Older adults   High Immersion (HMD)  Social   4 23 0.520 0.235 0.0490
## 71   Older adults   High Immersion (HMD)  Social   5 23 0.527 0.246 0.0513
## 72   Older adults   High Immersion (HMD)  Social   6 23 0.524 0.247 0.0515
```

2.5 Supplementary Figure 2. Plot of SCL time course

```
gscl<- ggplot(mseSCL, aes (x = bin, y=mean, fill =`Age Group`))+
  geom_line(lwd=0.75, aes(color=`Age Group`))+
    geom_point(size = 1.3,aes(color=`Age Group`))+
  geom_ribbon(aes(ymin = mean-se, ymax = mean+se, fill=`Age Group`), alpha = .2) +
   facet_grid(Content~Immersion)+
   ylab(" SCL range (%)")+
   theme_bw() +
  theme(legend.position = "bottom",
              axis.title = element_text(size=14),
        axis.text = element_text(size=12),
        legend.text = element_text(size = 12),
        legend.title = element_text(size = 14),
        strip.text = element_text(size=12.5),
        panel.grid.major = element_blank(), panel.grid.minor = element_blank(),
panel.background = element_blank(), axis.line = element_line(colour = "black"))+
  scale_color_manual(values = c("#000271", "#E70007"))+
  scale_fill_manual(values = c("#000271", "#E70007"))+
  scale_x_continuous(name ="Time bins (20 seconds each)" , breaks=seq(1, 6, 1))

gscl
```

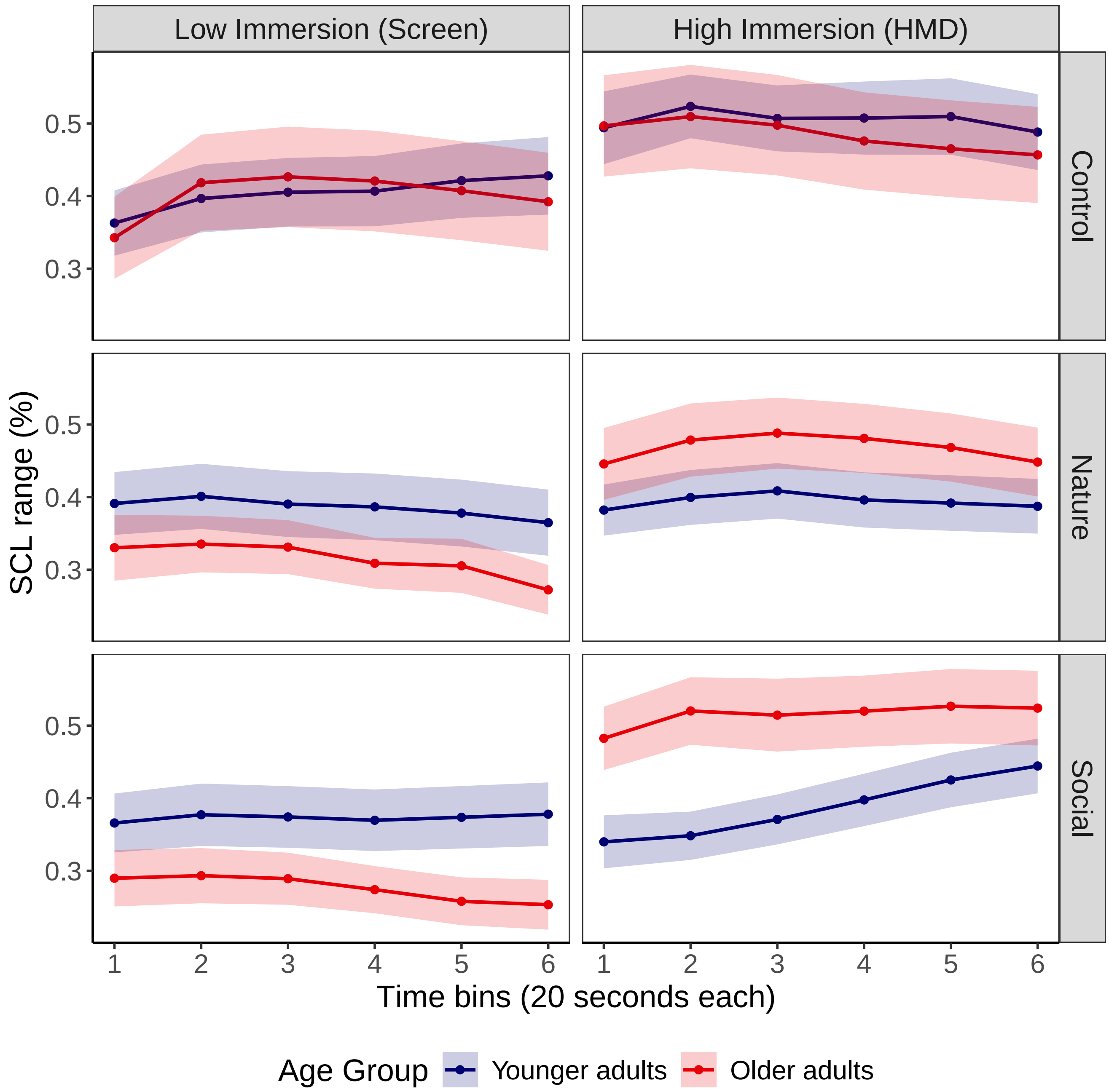

2.6 GCA on SCL - Results

```
mSCL0 <- lmer(value ~ GroupS*ImmersionS*ContentS+
                (1|Sujet),
                      control= lmerControl(optimizer="bobyqa"),
                      na.action = na.exclude,
                      data=SCL_long,
                      REML = F)


mSCL1 <- lmer(value ~ (ot1)*GroupS*ImmersionS*ContentS+
                (ot1||Sujet)+
```

```
                (ot1||Sujet:ImmersionS:ContentS),
                      control= lmerControl(optimizer="bobyqa"),
                      na.action = na.exclude,
                      data=SCL_long,
                      REML = F)


mSCL2 <- lmer(value ~ (ot1+ot2)*GroupS*ImmersionS*ContentS+
                (ot1+ot2||Sujet)+
                (ot1+ot2||Sujet:ImmersionS:ContentS),
                      control= lmerControl(optimizer="bobyqa"),
                      na.action = na.exclude,
                      data=SCL_long,
                      REML = F)
```

2.7 Supplementary Table 10. Models Comparisons

```
anova(mSCL0, mSCL1, mSCL2)
```

```
## Data: SCL_long
## Models:
## mSCL0: value ~ GroupS * ImmersionS * ContentS + (1 | Sujet)
## mSCL1: value ~ (ot1) * GroupS * ImmersionS * ContentS + (ot1 || Sujet) + (ot1 || Sujet:ImmersionS:Conte
## mSCL2: value ~ (ot1 + ot2) * GroupS * ImmersionS * ContentS + (ot1 + ot2 || Sujet) + (ot1 + ot2 || Suje
##       npar   AIC   BIC logLik deviance Chisq Df          Pr(>Chisq)
## mSCL0   14  -352  -273    190     -380
## mSCL1   29 -4596 -4432   2327    -4654  4274 15 <0.0000000000000002 ***
## mSCL2   43 -4951 -4708   2519    -5037   383 14 <0.0000000000000002 ***
## ---
## Signif. codes:  0 '***' 0.001 '**' 0.01 '*' 0.05 '.' 0.1 ' ' 1
```

Although the fourth model appeared significant, due to difficulties of this model to converge (warning about the singular fit) we prefer to be conservative and examine effects within the third model (mSCL3) containing first, second and third level polynomials.

2.8 Supplementary Table 11. Results of the selected model

```
anova(mSCL2)
```

```
## Type III Analysis of Variance Table with Satterthwaite's method
##                                Sum Sq Mean Sq NumDF DenDF F value   Pr(>F)
## ot1                           0.00007 0.00007     1    58    0.07  0.78786
## ot2                           0.01793 0.01793     1    58   18.48 0.000067 ***
## GroupS                        0.00000 0.00000     1    58    0.00  0.96781
## ImmersionS                    0.01723 0.01723     1   290   17.75 0.000034 ***
## ContentS                      0.00536 0.00268     2   290    2.76  0.06483 .
## ot1:GroupS                    0.00201 0.00201     1    58    2.07  0.15590
## ot2:GroupS                    0.00421 0.00421     1    58    4.34  0.04161 *
## ot1:ImmersionS                0.00126 0.00126     1   290    1.30  0.25523
## ot2:ImmersionS                0.00023 0.00023     1   290    0.24  0.62658
## GroupS:ImmersionS             0.00585 0.00585     1   290    6.03  0.01468 *
```

```
## ot1:ContentS                   0.00576 0.00288     2   290    2.97  0.05295 .
## ot2:ContentS                   0.00800 0.00400     2   290    4.12  0.01719 *
## GroupS:ContentS                0.00021 0.00010     2   290    0.11  0.89906
## ImmersionS:ContentS            0.00063 0.00032     2   290    0.33  0.72209
## ot1:GroupS:ImmersionS          0.00002 0.00002     1   290    0.02  0.87899
## ot2:GroupS:ImmersionS          0.00051 0.00051     1   290    0.53  0.46793
## ot1:GroupS:ContentS            0.00138 0.00069     2   290    0.71  0.49115
## ot2:GroupS:ContentS            0.00007 0.00004     2   290    0.04  0.96222
## ot1:ImmersionS:ContentS        0.01632 0.00816     2   290    8.41  0.00028 ***
## ot2:ImmersionS:ContentS        0.00425 0.00212     2   290    2.19  0.11387
## GroupS:ImmersionS:ContentS     0.00442 0.00221     2   290    2.28  0.10459
## ot1:GroupS:ImmersionS:ContentS 0.00045 0.00022     2   290    0.23  0.79502
## ot2:GroupS:ImmersionS:ContentS 0.00640 0.00320     2   290    3.30  0.03842 *
## ---
## Signif. codes:  0 '***' 0.001 '**' 0.01 '*' 0.05 '.' 0.1 ' ' 1
```

2.9 Supplementary Table 12. Estimates for significant effects and interactions

```
summary(mSCL2)
```

```
## Linear mixed model fit by maximum likelihood . t-tests use Satterthwaite's
##   method [lmerModLmerTest]
## Formula: value ~ (ot1 + ot2) * GroupS * ImmersionS * ContentS + (ot1 +
##     ot2 || Sujet) + (ot1 + ot2 || Sujet:ImmersionS:ContentS)
##    Data: SCL_long
## Control: lmerControl(optimizer = "bobyqa")
##
##      AIC      BIC   logLik deviance df.resid
##    -4951    -4708     2519    -5037     2045
##
## Scaled residuals:
##    Min     1Q Median     3Q    Max
## -6.179 -0.203 -0.012  0.194  9.752
##
## Random effects:
##  Groups                     Name        Variance Std.Dev.
##  Sujet.ImmersionS.ContentS   ot2         0.829230 0.9106
##  Sujet.ImmersionS.ContentS.1 ot1         5.403349 2.3245
##  Sujet.ImmersionS.ContentS.2 (Intercept) 0.046784 0.2163
##  Sujet                      ot2         0.124028 0.3522
##  Sujet.1                    ot1         1.366309 1.1689
##  Sujet.2                    (Intercept) 0.011431 0.1069
##  Residual                               0.000971 0.0312
## Number of obs: 2088, groups:  Sujet:ImmersionS:ContentS, 348; Sujet, 58
##
## Fixed effects:
##                                                   Estimate Std. Error
## (Intercept)                                       0.408889   0.018623
## ot1                                               0.055253   0.204562
## ot2                                              -0.325450   0.075746
## GroupS                                            0.000761   0.018623
## ImmersionS                                        0.050017   0.011872
## ContentS.Nature-intercept                        -0.018425   0.016789
## ContentS.Social-intercept                        -0.021006   0.016789
```

```
## ot1:GroupS                                   -0.294125   0.204562
## ot2:GroupS                                   -0.157805   0.075746
## ot1:ImmersionS                                0.150388   0.131283
## ot2:ImmersionS                                0.028669   0.059190
## GroupS:ImmersionS                             0.029177   0.011872
## ot1:ContentS.Nature-intercept                -0.413347   0.185662
## ot1:ContentS.Social-intercept                 0.365846   0.185662
## ot2:ContentS.Nature-intercept                -0.087053   0.083707
## ot2:ContentS.Social-intercept                 0.237513   0.083707
## GroupS:ContentS.Nature-intercept             -0.000111   0.016789
## GroupS:ContentS.Social-intercept              0.006763   0.016789
## ImmersionS:ContentS.Nature-intercept         -0.009170   0.016789
## ImmersionS:ContentS.Social-intercept          0.013231   0.016789
## ot1:GroupS:ImmersionS                        -0.019975   0.131283
## ot2:GroupS:ImmersionS                         0.042805   0.059190
## ot1:GroupS:ContentS.Nature-intercept          0.175610   0.185662
## ot1:GroupS:ContentS.Social-intercept         -0.204958   0.185662
## ot2:GroupS:ContentS.Nature-intercept          0.019367   0.083707
## ot2:GroupS:ContentS.Social-intercept          0.001429   0.083707
## ot1:ImmersionS:ContentS.Nature-intercept      0.168962   0.185662
## ot1:ImmersionS:ContentS.Social-intercept      0.558375   0.185662
## ot2:ImmersionS:ContentS.Nature-intercept     -0.140922   0.083707
## ot2:ImmersionS:ContentS.Social-intercept     -0.019615   0.083707
## GroupS:ImmersionS:ContentS.Nature-intercept   0.007216   0.016789
## GroupS:ImmersionS:ContentS.Social-intercept   0.026772   0.016789
## ot1:GroupS:ImmersionS:ContentS.Nature-intercept   0.121142   0.185662
## ot1:GroupS:ImmersionS:ContentS.Social-intercept  -0.089956   0.185662
## ot2:GroupS:ImmersionS:ContentS.Nature-intercept  -0.098612   0.083707
## ot2:GroupS:ImmersionS:ContentS.Social-intercept  -0.116075   0.083707
##                                                        df t value
## (Intercept)                                     58.000033   21.96
## ot1                                             58.000324    0.27
## ot2                                             58.000076   -4.30
## GroupS                                          58.000033    0.04
## ImmersionS                                     289.999908    4.21
## ContentS.Nature-intercept                      289.999916   -1.10
## ContentS.Social-intercept                      289.999916   -1.25
## ot1:GroupS                                      58.000324   -1.44
## ot2:GroupS                                      58.000076   -2.08
## ot1:ImmersionS                                 289.999202    1.15
## ot2:ImmersionS                                 290.000435    0.48
## GroupS:ImmersionS                              289.999908    2.46
## ot1:ContentS.Nature-intercept                  289.999202   -2.23
## ot1:ContentS.Social-intercept                  289.999202    1.97
## ot2:ContentS.Nature-intercept                  290.000435   -1.04
## ot2:ContentS.Social-intercept                  290.000435    2.84
## GroupS:ContentS.Nature-intercept               289.999916   -0.01
## GroupS:ContentS.Social-intercept               289.999916    0.40
## ImmersionS:ContentS.Nature-intercept           289.999916   -0.55
## ImmersionS:ContentS.Social-intercept           289.999916    0.79
## ot1:GroupS:ImmersionS                          289.999202   -0.15
## ot2:GroupS:ImmersionS                          290.000435    0.72
## ot1:GroupS:ContentS.Nature-intercept           289.999202    0.95
## ot1:GroupS:ContentS.Social-intercept           289.999202   -1.10
## ot2:GroupS:ContentS.Nature-intercept           290.000435    0.23
## ot2:GroupS:ContentS.Social-intercept           290.000435    0.02
```

```
## ot1:ImmersionS:ContentS.Nature-intercept        289.999202    0.91
## ot1:ImmersionS:ContentS.Social-intercept        289.999202    3.01
## ot2:ImmersionS:ContentS.Nature-intercept        290.000435   -1.68
## ot2:ImmersionS:ContentS.Social-intercept        290.000435   -0.23
## GroupS:ImmersionS:ContentS.Nature-intercept     289.999916    0.43
## GroupS:ImmersionS:ContentS.Social-intercept     289.999916    1.59
## ot1:GroupS:ImmersionS:ContentS.Nature-intercept 289.999202    0.65
## ot1:GroupS:ImmersionS:ContentS.Social-intercept 289.999202   -0.48
## ot2:GroupS:ImmersionS:ContentS.Nature-intercept 290.000435   -1.18
## ot2:GroupS:ImmersionS:ContentS.Social-intercept 290.000435   -1.39
##                                                             Pr(>|t|)
## (Intercept)                                     < 0.0000000000000002 ***
## ot1                                                           0.7880
## ot2                                                         0.000067 ***
## GroupS                                                        0.9675
## ImmersionS                                                  0.000034 ***
## ContentS.Nature-intercept                                     0.2734
## ContentS.Social-intercept                                     0.2119
## ot1:GroupS                                                    0.1559
## ot2:GroupS                                                    0.0416 *
## ot1:ImmersionS                                                0.2529
## ot2:ImmersionS                                                0.6285
## GroupS:ImmersionS                                             0.0146 *
## ot1:ContentS.Nature-intercept                                 0.0268 *
## ot1:ContentS.Social-intercept                                 0.0497 *
## ot2:ContentS.Nature-intercept                                 0.2992
## ot2:ContentS.Social-intercept                                 0.0049 **
## GroupS:ContentS.Nature-intercept                              0.9947
## GroupS:ContentS.Social-intercept                              0.6874
## ImmersionS:ContentS.Nature-intercept                          0.5854
## ImmersionS:ContentS.Social-intercept                          0.4313
## ot1:GroupS:ImmersionS                                         0.8792
## ot2:GroupS:ImmersionS                                         0.4702
## ot1:GroupS:ContentS.Nature-intercept                          0.3450
## ot1:GroupS:ContentS.Social-intercept                          0.2705
## ot2:GroupS:ContentS.Nature-intercept                          0.8172
## ot2:GroupS:ContentS.Social-intercept                          0.9864
## ot1:ImmersionS:ContentS.Nature-intercept                      0.3636
## ot1:ImmersionS:ContentS.Social-intercept                      0.0029 **
## ot2:ImmersionS:ContentS.Nature-intercept                      0.0934 .
## ot2:ImmersionS:ContentS.Social-intercept                      0.8149
## GroupS:ImmersionS:ContentS.Nature-intercept                   0.6676
## GroupS:ImmersionS:ContentS.Social-intercept                   0.1119
## ot1:GroupS:ImmersionS:ContentS.Nature-intercept               0.5146
## ot1:GroupS:ImmersionS:ContentS.Social-intercept               0.6284
## ot2:GroupS:ImmersionS:ContentS.Nature-intercept               0.2397
## ot2:GroupS:ImmersionS:ContentS.Social-intercept               0.1666
## ---
## Signif. codes:  0 '***' 0.001 '**' 0.01 '*' 0.05 '.' 0.1 ' ' 1

##
## Correlation matrix not shown by default, as p = 36 > 12.
## Use print(x, correlation=TRUE)  or
##     vcov(x)        if you need it
```

2.9.1 GCA on SCL for SC

```
mSCLSC <- lmer(value ~ (ot1+ot2)*GroupS*ContentS+
                (ot1+ot2||Sujet)+
                (ot1+ot2||Sujet:ContentS),
                      control= lmerControl(optimizer="bobyqa"),
                      na.action = na.exclude,
                      data=subset(HR_long, Immersion=="Low Immersion (Screen)"),
                      REML = F)
```

```
anova(mSCLSC)
```

2.9.1.1 Supplementary Table 13. Results of the model

```
## Type III Analysis of Variance Table with Satterthwaite's method
##                     Sum Sq Mean Sq NumDF DenDF F value  Pr(>F)
## ot1                   44.5    44.5     1    58   13.88 0.00044 ***
## ot2                    4.9     4.9     1    58    1.52 0.22216
## GroupS                28.1    28.1     1    58    8.74 0.00449 **
## ContentS               7.3     3.6     2   116    1.14 0.32483
## ot1:GroupS            18.9    18.9     1    58    5.89 0.01839 *
## ot2:GroupS             1.6     1.6     1    58    0.50 0.48307
## ot1:ContentS           6.1     3.0     2   116    0.95 0.39075
## ot2:ContentS           3.1     1.5     2   116    0.48 0.62267
## GroupS:ContentS        0.0     0.0     2   116    0.01 0.99356
## ot1:GroupS:ContentS    1.3     0.6     2   116    0.20 0.81853
## ot2:GroupS:ContentS   13.5     6.7     2   116    2.10 0.12671
## ---
## Signif. codes:  0 '***' 0.001 '**' 0.01 '*' 0.05 '.' 0.1 ' ' 1
```

2.9.2 GCA on SCL for VR

```
mSCLVR <- lmer(value ~ (ot1+ot2)*GroupS*ContentS+
                (ot1+ot2||Sujet)+
                (ot1+ot2||Sujet:ContentS),
                      control= lmerControl(optimizer="bobyqa"),
                      na.action = na.exclude,
                      data=subset(HR_long, Immersion=="High Immersion (HMD)"),
                      REML = F)
```

```
anova(mSCLVR)
```

2.9.2.1 Supplementary Table 14. Results of the model

```
## Type III Analysis of Variance Table with Satterthwaite's method
##                     Sum Sq Mean Sq NumDF DenDF F value Pr(>F)
## ot1                   28.8   28.83     1    58    9.96 0.0025 **
```

```
## ot2                   1.5    1.54     1   174    0.53 0.4666
## GroupS               27.4   27.43     1    58    9.48 0.0032 **
## ContentS             11.6    5.82     2   116    2.01 0.1386
## ot1:GroupS           10.1   10.14     1    58    3.50 0.0662 .
## ot2:GroupS            1.8    1.83     1   174    0.63 0.4279
## ot1:ContentS         20.3   10.14     2   116    3.50 0.0334 *
## ot2:ContentS         36.6   18.28     2   174    6.31 0.0023 **
## GroupS:ContentS       6.9    3.46     2   116    1.19 0.3068
## ot1:GroupS:ContentS   5.5    2.73     2   116    0.94 0.3921
## ot2:GroupS:ContentS   0.2    0.12     2   174    0.04 0.9580
## ---
## Signif. codes:  0 '***' 0.001 '**' 0.01 '*' 0.05 '.' 0.1 ' ' 1
```
